# Advances in magnetic affinity-based isolation/detection of exosomes for robust diagnostics

**DOI:** 10.1007/s00604-025-07048-6

**Published:** 2025-03-05

**Authors:** Veronika Solovicová, Anna Ďatková, Tomáš Bertók, Peter Kasák, Alica Vikartovská, Lenka Lorencová, Jan Tkac

**Affiliations:** 1 Institute of Chemistry, Slovak Academy of Sciences, Dúbravská cesta 5807/9, 845 38 Bratislava, Slovak Republic; 2 Center for Advanced Materials, Qatar University, P.O. Box 2713, Doha, Qatar

**Keywords:** Cancer diagnostics, Biomarker discovery, Exosomes, Magnetic beads, Affinity capture

## Abstract

The review article provides a short introduction to exosomes with the focus to use exosomes as disease markers itself (i.e. their concentration or presence of some specific receptors) or a source of disease biomarkers such as proteins and metabolites. In detail, we are discussing various methods of exosome isolation and the main focus of the review paper is on affinity capture of exosomes, since some of them can be applied to the isolation of specific sub-populations of exosomes produced by some specific organs. The article provides a comprehensive overview of magnetic (bio)affinity capture applied to the detection of exosomes or exosomal cargo using different (bio)affinity capture ligands such as antibodies, DNA aptamers, peptides, glycan-based recognition, transferrin-based approaches, affinity based on recognition of phospholipids of exosomes and other approaches including electrostatic interactions. The review in detail provides key analytical and clinical parameters of such approaches in a form of an extensive table summarising outcomes published in the last two years (2023–2024). Finally, the review paper also provides conclusions sections discussing *pros* and *cons* of magnetic (bio)affinity capture for exosome isolation and/or determination  of exosomal content.

**Graphical abstract:**

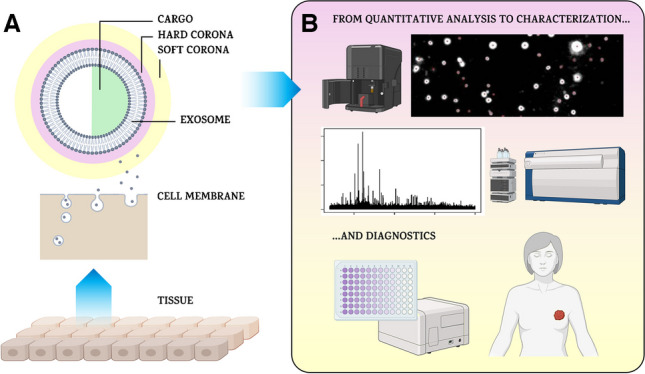

## Exosomes

**Fig. 1:**
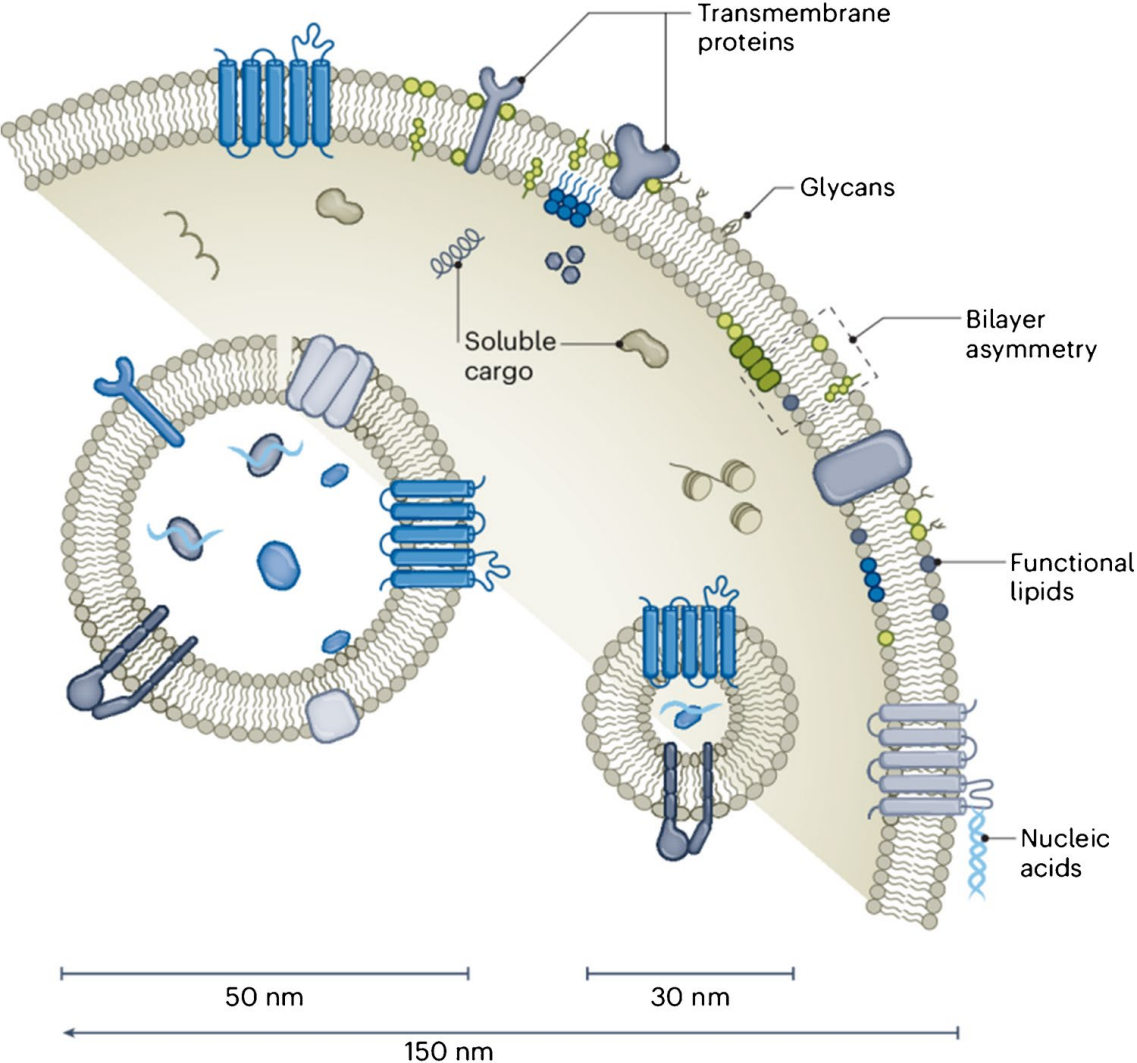
EVs exhibit major heterogeneity that currently limits their effective application in drug delivery. EVs are heterogeneous in size, from ~ 30 nm to more than 150 nm. The smallest and largest EVs are unlikely to exhibit similar functions due to large differences in their volume, surface area and composition (for example, membrane components and soluble cargo). Reproduced with permission from Springer Nature from ref. [1]

Exosomes belong to the group of extracellular vesicles (EVs), which are natural nano- and microparticles produced by different types of cells (Fig. 1) [1]. EVs are usually categorised into several groups depending on their size, i.e. exomeres (~ 35–50 nm, non-membranous particles), exosomes (~ 30–150 nm), microvesicles (~ 100–1,000 nm) and apoptotic bodies (~ 500–3,000 nm) (Table 1) [2, 3]. Exosomes as biological nanoparticles first described in 1985 as reticulocytes [4] and sometimes called also intraluminal vesicles are formed by an endosomal route from early endosomes and multivesicular bodies (MVBs) [5]. In contrast, EVs formed by outward budding from the plasma membrane are called ectosomes [6]. Exosomes are found in a variety of biological fluids, such as plasma, saliva, urine, amniotic fluid, synovial fluid, breast milk and even tears [7–11]. Exosomes consist of rich membranes containing a diverse range of biomolecules such as lipids, sterols, membrane proteins and glycans surrounding aqueous exosomal luminal cargo containing proteins, glycoproteins, nucleic acids, metabolites, cytokines and other functional biomolecules [1].

**Table 1:** Types of vesicles and their attributes [13, 16, 17]

Vesicles	Size	Markers	Biogenesis
Exomeres	~ 30—50 nm	unknown	unknown
Exosomes	30—150 nm	CD9, CD63, CD81, CD82, Tsg101, ALIX, flotillin, HSPs	inward budding of MVBs
Microvesicles (ectosomes)	100—1,000 nm	ARF6, Annexin A1, integrins, selectins	pinching off the membrane
Apoptotic bodies	50—5000 nm	phosphatidylserine, histones	formed from apoptotic cells

*Tsg101* tumour susceptibility gene 101 protein, *ALIX* apoptosis-linked gene 2 product interacting protein, *HSPs* heat shock proteins, *ARF6* ADP-ribosylation factor 6, *MVBs* multivesicular bodies, *CD* clusters of differentiation

**Fig. 2:**
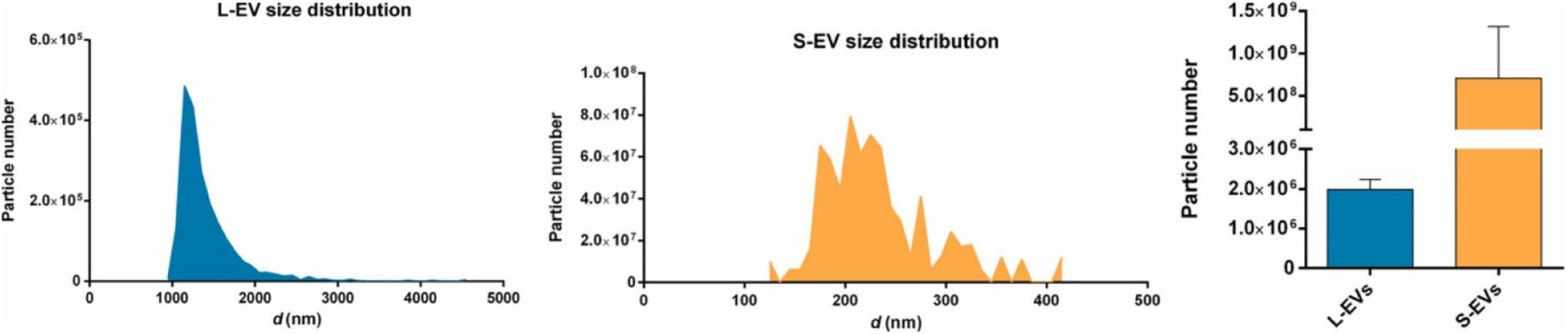
Characteristics of EVs. Representative size distribution for L-EV (microvesicles) (left) and S-EV (exosomes) (middle) with a direct comparison of L-EV *vs.* S-EV particle number in the analysed EV preparation. EVs were produced by a PC3 prostate cancer cell line. Reprinted from ref. [18], Copyright (2022), with permission from Elsevier

Thus, exosomes are a promising source of different types of biomarkers in so-called liquid biopsy approaches and other diagnostic applications [3]. Among these biomarkers, micro RNA (miRNAs) within an exosome are of a great importance [12]. Surface-exposed biomarkers include Rab7, Rab8, Rab11, Rab27, and Rab35 (Rabs are small guanine triphosphatases) regulating the exosomes pathway, SNARE proteins (SNARE = *N*-ethylmaleimide-sensitive factor activating protein receptors) driving the fusion of MVBs and the cellular plasma and CD9, CD63, CD81, Alix, and Tsg101 – often used to selectively detect and fish-out exosomes from a complex biological sample [13]. In addition to these molecules, 5–70 nm thick corona layer is formed in a proximity to the exosome surface, being an integral part of these biological vesicles, possibly being affected by different conditions and causing also a wide range of possible diameters [14]. The size of exosomes also influences the number of proteins present on the exosomal membrane (Fig. 1 and Table 2). For example, the exosome with the size of 100 nm has 37-fold larger volume compared to 30 nm exosomes and potentially containing thousand more proteins and other biomolecules [1]. The concentration of EVs in biofluids exhibits a log-linear decrease with increasing their size (Fig. 2 right) with the size distribution for exosomes (S-EVs, i.e. small EVs) (Fig. 2 middle) and microvesicles (L-EVs, i.e. large EVs) shown in Fig. 2 left [1]. The concentration of exosomes (~ 100 nm) isolated from healthy human serum is approximately 7 × 10^8^ exosomes per mL with their concentration significantly increased in case of individuals with oncological diseases (~ 10^10^–10^11^ exosomes per mL) [15].

**Table 2:** The relative volume, surface area and number of membrane proteins scale for a given vesicle size [1]

Diameter (nm)	Relative volume	Relative surface area	Number of proteins
30	1 ×	1 ×	100
50	5 ×	3 ×	300
100	37 ×	11 ×	1,100
150	125 ×	25 ×	2,500

Exosomes are involved in numerous biological functions including signal transduction in intercellular communication, extracellular matrix remodelling, supressing anti-tumour immunity, inducing angiogenesis, etc. [1, 3]. There is an increasing evidence for the exosomes to form a pre-metastatic niche during tumorigenesis, possibly transferring a bio-information encoded in different biopolymers or even metabolism-associated receptors to fuel a cancer metabolism or remodelling the extracellular matrix [19, 20]. Exosomes are also responsible for an immune cell suppression and epithelial-to-mesenchymal transition [21]. Combination of exosomes and nanoparticles has been shown to be a promising tool not only in diagnostics, but targeted drug delivery as well—a concept recognised and popularised as early as by the end of the nineteenth century by Paul Ehrlich and his Zauberkugeln (magic bullets) [22]. Mesenchymal stem cells-derived exosomes are commonly used to treat inflammation and for drug delivery, while dendritic cells-derived ones can be used to induce the inflammation against cancer cells [13]. Generally, constructed by an iterative physical extrusion or freeze/thaw cycles, exosomes-biomimetic nanoparticles are increasing considerable attention [23]. As for the diagnostic applications, nanomaterials can be used: (i) as a capture probe due to an increase in surface area for accommodation of affinity ligands or (ii) as signal transducers and/or amplifiers [24].

Oncology needs more accurate and tissue-specific biomarkers for early cancer detection and prognostic purposes as well. For this purpose, non-invasive sample collection and disease-specificity is of great importance [25]. Compliance with these new liquid biopsy methods with the established ones needs to be addressed as well, so the benefit is clear. For instance a laboratory test for an early prostate cancer detection, which in combination with magnetic resonance imaging (MRI) may significantly lower the amount of unnecessary (early) prostate biopsies leading to overdiagnosis/overtreatment or creating a barrier between patients and clinicians for further monitoring due to an unpleasant experience [26]. ExoDx Prostate (IntelliScore) “EPI” as a non-invasive urine CE-IVD (CE certified in vitro diagnostic device) test utilizing exosomal RNA showed a superior performance to two commonly used multiparametric risk calculators, i.e. Prostate Cancer Prevention Trial and European Randomised Study of Screening for Prostate Cancer risk calculators [27]. The importance of clinical validations of exosome-based diagnostic tests using real life samples with robust background data is eminent, as these will subsequently lead to new diagnostic approaches on the market. For routine use of tumour-derived exosome biomarkers, robust and reproducible pre-analytical protocols need to be developed to ensure a high-quality input material and an inter-batch comparability during a clinical translation process [28, 29]. Clinical usefulness of novel biomarkers needs to be proved by running clinical validation studies, i.e. analysis of cancerous samples and samples from healthy controls (HC). Evaluation of clinical validation study is usually performed using a Receiver Operating Curve (ROC) from which clinical parameters such as accuracy, sensitivity (correctly identified disease samples), specificity (correct identification of HC), AUC value (Area Under Curve, ROC curve) and other parameters. In the following text, we will refer to AUC values obtained from ROC curve, which in ideal case should receive value of 1.00 (a perfect discrimination power), but values above 0.80 still provide a good discrimination power of the biomarker.

Furthermore, novel biomarkers can be investigated in clinical trials for regulatory purposes. In the publication published by Rezaie et al. in 2022, there is stated that exosomes have been investigating in 116 clinical studies to reveal their potential as disease biomarkers (50.0% studies), from which 74.1% of (43 trials) were focused on investigation of exosomes as a source of cancer biomarkers [13]. Cancer-related clinical studies were focused on exosomal biomarkers for the following types of cancer: lung (13), breast (6), colorectal (4), pancreatic (4), thyroid (4), prostate (3), etc. [13]. Exosome-based diagnostics of prostate cancer [30] and lung cancer [31] were in details recently reviewed. In a more recent study published by Dilsiz [2] in 2024 it is stated that exosomes have been used in more than 400 clinical studies for various diseases including therapeutics and disease biomarkers. A list of cancerous exosomal biomarkers, which could be applied for disease diagnostics or disease prognosis is shown in Table 3.

**Table 3:** A list of exosomal cancerous biomarkers applied in disease diagnostics or disease prognosis [17]

Biomarker	Source of EVs	Type of cancer	Application
CD147	S	CRC	diagnostics & prognosis
EpCAM	P	CRC	diagnostics
LGALS3BP	S	endometrial	diagnostics
EGFRvIII	P	glioblastoma	prognosis
TYRP2	P	glioblastoma	diagnostics
VLA-4	P	melanoma	diagnostics
HSP70	P	melanoma	prognosis
PD-L1	S	melanoma	prognosis
MDA-9, GRP78	S	melanoma	diagnostics
LRG1	P	NSCLC	diagnostics
Glypican-1	S	PaCa	diagnostics
PDCD6IP, FASN, XPO1, ENO1	P	PCa	diagnostics

*S* human blood serum; *P* human blood plasma; *EGFRvIII* = *EGFR* epidermal growth factor receptor; *EpCAM *epithelial cell adhesion molecule; *HSP* heat shock protein; *LGALS*3*BP* lectin galactoside-binding soluble 3 binding protein (galectin 3 binding protein); *LRG*1 leucine-rich alpha-2-glycoprotein 1; *MDA*−9 melanoma differentiation associated gene-9 (syntenin1 or syndecan binding protein); *GRP*78 glucose regulated protein 78; *PDCD*6*IP* programmed cell death 6 interacting protein; *FASN *fatty acid synthase; *XPO*1 exportin 1; *ENO*1 enolase 1; *PD-L*1 programmed death-ligand 1; *TYRP*2 tyrosinase-related protein 2; *VLA*−4 very late antigen-4 (integrin α4β1); *CRC* colorectal cancer; *PaCa* pancreatic cancer; *PCa* prostate cancer; *NSCLC* non-small cell lung cancer

**Fig. 3:**
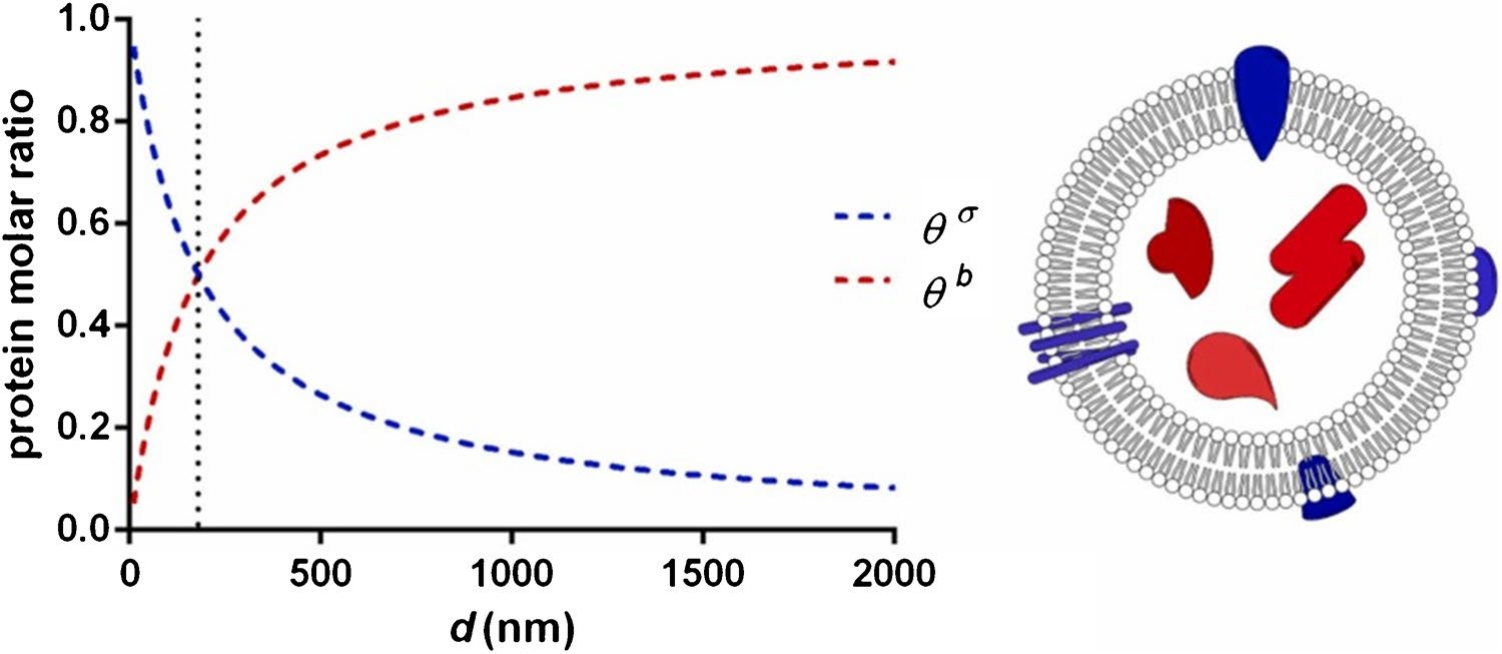
Protein molar partition between the membrane of EVs (*θ*^*σ*^) and luminal part of EVs (*θ*^*b*^) as a function of EV radius. Reprinted from ref. [18], Copyright (2022), with permission from Elsevier

**Fig. 4:**
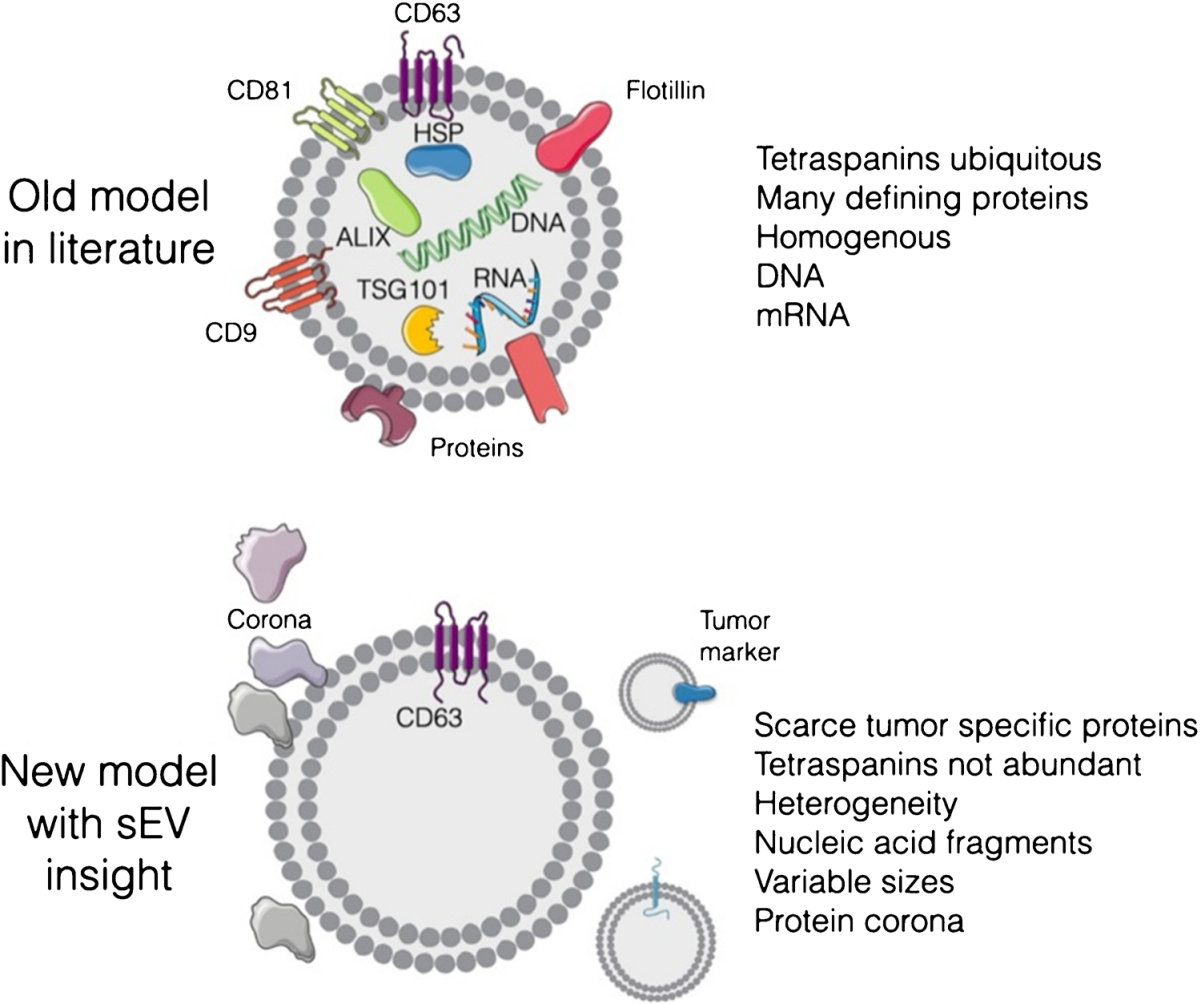
New insights into the composition of EVs. An old model describes that EVs contain a large number of defining ubiquitous proteins and nucleic acids. In reality, studies focused on analysis of single EVs have shown much less abundant proteins at the single EV level, scarcity of tumor-defining biomarkers, and a protein corona. Reprinted from ref. [16]. Copyright (2022), with permission from Elsevier

There is, however, a significant heterogeneity of exosomes since different sources of exosomes (i.e. cells) produce exosomes with different composition and function (i.e. inter-subpopulation heterogeneity), but also the same source can produce different exosomes within subpopulations (i.e. intra-subpopulation heterogeneity) [1]. As a consequence, heterogeneity in molecular composition and other attributes of exosomes result in the diversity in spatial distribution and thus biological response of exosomes [32]. Typical attributes of exosomes can be divided into two main groups [1, 33, 34]: 1. physical parameters (size, density, viscoelasticity) and 2. molecular composition, i.e. a presence of specific receptors on the EVs membrane. Depending on the size and composition of EVs in general, such particles can have highly specific roles [1, 35]. EVs with difference in their size, but produced by the same source, could exhibit differential biological functions, when, for example the size of EVs significantly influences cellular uptake of EVs [36]. It seems that when talking about EVs, the size matters, for small EVs with the size up to 180 nm, membrane-associated proteins are prevalent, while large EVs ( microvesicles) contain more luminal proteins than membrane proteins and thus the luminal cargo dominate their function (Fig. 3) [18]. New insights suggest, when analysing single EVs that EVs are only sparsely covered by receptors with presence of corona [16]. Thus, the composition of exosomes is reconsidered with a shift from an “old model” to a “new model” of the composition of EVs (Fig. 4), but more studies are needed to be performed with the focus on analysis of individual EVs. The main consequence when taking into account a “new model” of the composition of EVs might be the fact that most likely the composition of corona could be a source specific, i.e. depending on the composition of a biofluid the EVs are isolated from (serum, plasma, cell line cultivation media, urine, etc.). Thus, the (bio)affinity capture reagents working for isolation of EVs from one source might not work as effectively for isolation of EVs from the other source and vice versa.

## Isolation of exosomes

The true potential of exosomes as a source of biomarkers can be revealed only in case we are able to isolate exosomes with a high purity from other bodily fluids including cells, apoptotic bodies, microvesicles, proteins, and lipids. Isolation of exosomes should be performed in a simple and quick way with high throughput, recovery rate and at an affordable cost. Typical exosome isolation procedures include ultracentrifugation (UC), ultrafiltration (UF), (bio)affinity capture (AC), size-exclusion chromatography (SEC), polyethylene glycol (PEG)-based precipitation, microfluidics-based techniques (MF) having advantages and limitations. Therefore, an effective and reliable isolation of exosomes is still a significant challenge and there is recommendation to combine at least two different isolation procedures to isolate pure exosomes. Moreover, there are several commercially available kits available for exosome isolation [2]. Once exosomes are isolated, it is very important to keep them in the buffer, which can guarantee long stability upon storage with PBS buffer containing 25 mM trehalose being such a buffer according to the recent publication [37].

### Isolation of exosomes from various sources

The first step in the isolation of exosomes from cell cultures is removal of cells and their debris by centrifugation at 300 g and then at 2,000 g for 10 min at 4 °C. Then, the isolation of choice can be performed and exosomes should be stored at −20 °C or at −80 °C until they are needed for further experiments. It is recommended to purify exosomes from cell cultures grown in a chemically defined medium to limit presence of contaminating serum exosomes, proteins, and lipoproteins [2].

Isolation of exosomes from urine also starts with removal of cells and cell debris, which can be present in urine using centrifugation at 300 g and then at 2,000 g for 10 min at 4 °C. Then, the further procedure as described above for isolation of exosomes from various cell cultures is performed [2].

Blood is the most frequently studied source of biomarkers and exosomes. Exosomes can be isolated both from blood plasma and serum, but isolation of exosomes from plasma is preferred (62% cases) [2]. The main reason behind that is the fact that platelet aggregation used for serum preparation can trap exosomes within the aggregates limiting the yield of exosomes isolated. Presence of reddish colour in plasma or serum indicate that the process was non optimal with red blood cells hemolysed. Serum or plasma should be yellow in color and removal of cells or cell debris should be done by centrifugation at 2,000 g for 10 min at 4 °C and during centrifugation plasma/serum should also contain the protease/phosphatase inhibitor cocktail. Processed serum/plasma samples contain 10^8^–10^10^ exosomes*/*mL; 10^6^ large EVs per mL compared to 10^16^ lipoprotein particles*/*mL [2]. Finally, plasma/serum samples are aliquoted and stored at −80 °C for further analysis.

A fundamental understanding of the roles of exosomes related to diseases requires isolating them from tissue samples. The first step would be to cut the tissue samples into small pieces (~ 2 mm sections) using tissue homogenizers, followed by an incubation of the samples for 30 min at 37 °C using a shaker with protease (i.e. collagenase) and phosphatase inhibitors. The tissue samples are then centrifuged at 500 g for 10 min (removal of cells), followed by centrifugation at 10,000 *g* for 40 min (removal of microvesicles) and finally filtered using a 0.8 μm filter. Exosomes are then isolated using an exosome purification procedure of choice and finally stored at −80 °C for further analysis [2].

### Ultracentrifugation (UC)

UC is the gold standard for exosome purification (60% of the cases) [2], a method based on sedimentation (combination of the exosome size and density) during centrifugation at a high rotation speed (~ 100,000–150,000 g) at 4 °C. Finally, exosomes are stored at −80 °C for further analysis. UC is simple to perform, time-consuming and able to process large volume of samples (up to 25 mL), but specialised infrastructure is needed. The main drawback of the method is a high shear force applied during the process, what can disrupt the structure of exosomes. There are two different types of UC, i.e. differential UC and density gradient UC.

#### Differential UC (DUC)

DUC or differential velocity centrifugation or as simply called ultracentrifugation, consists of several rounds of centrifugation with a gradually increasing rotation speed with a total time up to 12 h: 500 g (~ 1 h); 3,000 g (~ 1 h); 16,000 g (~ 1 h) and finally 100,000–150,000 g (~ 1–6 h). The exosomes are present in the pellet, washed by PBS and stored in storage buffer at −80 °C for further analysis. The DUC process requires active involvement of a user to separate supernatant from pellets and for starting new cycles. Furthermore, a loss of exosomes can occur during repeated supernatant removal. The final product by implementing DUC contains ~ 70% of exosomes (50–150 nm) and only a limited amount of EVs having size below 50 nm and above 150 nm [2]. DUC is a labor-intensive and has to be optimised based on the rotor type and its parameters on one side and the types of the sample used for exosome isolation. On the other hand, DUC offers purification of several samples at once and do not require use of other chemicals. Quite high sheer force applied during DUC can damage exosomes or induce their aggregation. DUC is recommended for purification of exosomes from complex samples with a high degree of heterogeneity [2].

#### Density gradient UC (DGUC)

The DGUC also known as isopycnic UC employs a density gradient formed using different agents such as sucrose, iodixanol, and iohexol. The biocomponents including EVs move across the gradients formed in the centrifuge tubes during the process until they reach the section where the density of biocomponents/EVs is the same as the density of surrounding liquid. The DGUC separates particles according to their size and mass density in an effective way. The sample (typically ~ 1 mL) is loaded into the tube using a “bottom-up” or “top-down” approach, but in both cases the sample is loaded to the least dense gradient in the tube. Finally, the fractions (typically ~ 1 mL) must be carefully collected to do not disrupt the gradient. After the process, UC at 100,000 g for 2 h at 4 °C is still required. The exosome sample is then stored at −80 °C for subsequent analysis. DGUC isolates exosomes with a lower yield, but a higher purity compared to UC (DUC). Formation of a density gradient contributes towards the increase cost, time (~ 24 h) and a labour intensity of the process compared to UC (DUC). This is why, DGUC is not that frequently applied for exosome isolation [2].

### Size-based techniques

There are three different types of size-based techniques for exosome isolation including ultrafiltration, sequential filtering, and size-exclusion chromatography with a typical time to perform them in the range from 2 h to 4 h.

#### Ultrafiltration (UF)

UF also known as membrane filtration is based on utilisation of membrane filters with molecular weight cut-offs (MWCO) of 10 kDa, 50 kDa, and 100 kDa. In the first step, cells, cell debris and microparticles are separated using membrane filters with pore diameters of 0.1 μm, 0.22 μm, and 0.45 μm. Then, to remove proteins and other biocomponents, membrane filters with MWCO ranging from 5 kDa to 100 kDa are used. For further pre-concentration of exosomes UC (DUC) can be performed. UF separates exosomes using a membrane with the pore size lower than 100 nm and can be performed in several variations such as sequential filtration, centrifugal ultrafiltration, tandem filtration, and tangential flow filtration [2, 38].

Tandem filtration combines several filters in a single syringe, while sequential filtration requires several rounds of filtration with a distinct MWCO membrane used. In the centrifugal UF, the sample is forced through the membrane, while centrifuged. Tangential flow filtration is based on passing the samples tangentially to the membrane rather than by sample passing orthogonally to the membrane as discussed above for other UF methods. The main limitations of all UF methods include damage of exosomes during the process, adsorption of exosomes on the membranes (loss of yield) and clogging of the membrane (extended time of separation). Tangential follow filtration is a gentler to the samples, larger volume of samples can be processed with higher reproducibility compared to UC, but the method takes longer time compared to other UF methods. In general UF based methods of exosome isolation are less complicated, faster and request less complex infrastructure compared to UC-based methods.

#### Size exclusion chromatography (SEC)

SEC or gel filtration is the gentlest chromatography method used for isolation of exosomes according to their size, providing a high yield while preserving integrity and biological function of exosomes. Sample is placed on the top of the stationary phase containing porous material such as agarose, polyacrylamide or dextran with small biocomponents (proteins) passing quickly and exosomes retained for a longer time within the matrix. The SEC process is influenced by a wide range of parameters including dimension of the column, type and packaging density of porous material, flow of mobile phase and the overall volume of the column. SEC is a quick process, but additional purification process such as UC or UF are usually needed to remove impurities such as lipoproteins and proteins, increasing purity of exosomes [2]. The SEC is a cost-effective, reproducible process of exosome purification preserving their integrity and a biological function. Furthermore, the method can work with a minute amount of sample (down to 20 μL) in a short time (10–20 min).

### (Bio)affinity capture (AC)

AC utilises (bio)affinity-based reagents such as antibodies, aptamers or other affinity ligands (i.e. transferrin), which are usually attached to the surface of magnetic beads (MBs), made of different types of material (iron, neodymium or nickel based MBs). AC allows isolating exosomes based on presence of unique features on the exosomes (i.e. specific receptors) to separate exosomes into different subpopulations produced by different cell types and to study functional consequences of subtypes of exosomes or to apply exosome subpopulation in the biomarker discovery. The method also allows imaging of individual exosomes by visualisation of protein receptors on the membrane of exosomes. Furthermore, AC is a gentle method maintaining the function and structure of exosomes. Typical membrane receptors targeted in the separation include proteins such as clusters of differentiation (i.e. CD9, CD56, CD63, CD81, CD82, CD91, CD105, CD147, and CD151) and other proteins such as Apoptosis-Linked gene 2-interacting protein X (ALIX), and Epithelial Cell Adhesion Molecule (Ep-CAM), etc. While CDs, ALIX, Rabs, SNAREs, and Tsg101 proteins are present on exosomes produced by a variety of different cells [39], EpCAM receptor can be rather used for isolation of cancerous exosomes [40].

AC is performed by incubation of MBs with immobilised/attached (bio)affinity ligands with the sample, followed by magnetic separation using a permanent magnet (there are even ELISA based magnets allowing to work using ELISA plates) and wash of MBs to reduce non-specific binding. Then, exosome can be released from MBs using different types of reagents depending on the nature of affinity binding (i.e. change in pH, ionic strength, addition of an excessive amount of target molecules such as sugars or lipids, absence of bivalent ions or presence of chelating agents such as EDTA or other sophisticated tools described below). In order to eliminate protein aggregates or other large particles it is advised to combine the method with other pre-processing isolation methods such as SEC or a centrifugation. The AC method offers higher specificity of exosome isolation in comparison to UC, but with lower yield. The main drawbacks of the AC method include low volume of sample, which can be processed. It is needed to state also the obvious fact that with AC only subpopulations of exosomes with presence of specific receptors are isolated and not all types of exosomes. Other drawbacks of the method are quite expensive antibodies used for bioaffinity capture, what can be addressed using other types of affinity ligands such as DNA/RNA aptamers, lipids, transferrin, peptides or lectins (glycan recognised proteins) [41–52].

The main issue is non-specific binding, especially, when using complex samples, what should be resolved carefully. AC is a very useful method for isolating a particular subpopulation of exosomes that originate from a specific type of tissue cell and due to high specificity of the isolation method, AC protocol is an ideal method for exosome isolation for subsequent specific diagnostics of diseases.

There are new affinity based methods for isolation of EVs based on affinity towards phospholipids, what is an interesting approach rapidly evolving exhibiting a high specificity and membrane affinity [53]. The method is effective, flexible, simple, and controllable utilising a hydrophobic and other types of interactions [53].

### Precipitation method

Precipitation-based exosome isolation methods exploit chemical precipitation of exosomes using highly hydrophilic polymers such as polyethyleneglycol (PEG) [54] with M_w_ of ~ 6–8 kDa by overnight incubation at 4 °C. The method relies on removal of water molecules associated with negatively charged exosomes by binding water molecules to PEG. The final step is centrifugation of precipitated exosomes at 16,000 g for 20 min at 4 °C. The pellets of exosomes are then re-suspended in PBS and kept at −80 °C until further analysis. There are several companies offering commercially available kits for exosome isolation based on precipitation to make the process time effective and allowing working with a minute sample volume, but reliability and specificity of exosome isolation is highly variable and not always cost-effective. The protocol is easy, rapid (~ 2 h), do not require specialised equipment and do not destroy exosomes, but the product is with low purity. Furthermore, the method has limitations, when working with samples containing high protein content and contaminate the samples by the biopolymers used for precipitation. The precipitation-based purification of exosomes can be combined with filtration, centrifugation or gel filtration methods. Besides using highly hydrophilic polymers for precipitation of exosomes, for the same purpose also organic solvents, i.e. acetone can be used [54]. There are few companies selling exosome isolation kits based on precipitation including ExoQuick Plus (System Biosciences, USA), ExoEasy (Qiagen, The Netherlands) and Total Exosome Isolation kit (Invitrogen, USA) [2, 55].

### Microfluidics-based techniques (MF)

New advanced techniques using MF can be effectively applied for isolation of exosomes from samples with a minute volume in a time- and cost effective way, offering also highly accurate/reliable results [56]. MF is integrated with size-based and AC-based methods of separation [57–59]. MF systems integrate two or more devices/approaches to autonomously operate in parallel via a network of interconnected nano/micro-channels. Size-based separation of exosomes can be realised using various nano/micro-pillars, nano-/micro-pores or using acoustic waves. The purity of exosomes produced by such devices can be very high with the biological role of exosomes still preserved. The alternative way for purification of exosomes within MF is to use electric field since exosomes are negatively charged and can be separated from neutral and positively charged particles/biomolecules. MF has some disadvantages for exosome isolation including requirement for specialised expensive equipment, low sampling efficiency, frequent channel blockage, and requirement for high affinity and specificity antibodies. Microfluidics can be also used to isolate exosomes with different density of receptors expressed, based on the density of expressed exosomal receptors using MBs labelled with antibodies against such receptors, i.e. anti-PD-L1 antibodies, since higher receptor density expressing exosomes bind higher amount of MBs affecting magnetic properties of such particles, what is used for separation [60]. Isolation of exosomes using microfluidic-based devices can be performed in a form of a service provided by the companies Creative Biostructure and Creative Biolabs (using several trapping principles), but we have not identified any company selling the microfluidics-based isolation of exosomes. The main reason might be a requirement for a trained personnel (Table 4).

**Table 4:** A summary of exosome isolation methods and their attributes [2, 38, 61, 63]

Method	Yield	Purity	Sample volume	Biol. activity	Time	Advantages	Disadvantages
DUC	+ / + +	+ / + +	M—L	+ +	up to 12 h	multiple samples at once; standardised	expensive equipment needed; time-consuming; labour-intensive; exosome damage; presence of contaminants; low throughput
DGUC	+ +	+ + / + + +	M—L	+ +	up to 24 h	standardised; potential subtype isolation	expensive equipment needed; time-consuming; labour-intensive; exosome damage; low throughput
UF	+	+	S—M	+	~ 2 h	easy to perform; frequently used	exosome damage; block of pores; adsorption of exosomes
TFF	+ +	+ +	L	+ + +	~ 4 h	easy to perform	contamination from the same sized other particles
SEC	+ +	+ +	S—M	+ + +	~ 10–20 min	cost-effective; effective in removing proteins; scalable; medium to high throughput; easy to perform	complicated; labour-intensive
AC	+ +	+ + / + + +	S	+	~ 1 h	high specificity; easy to perform; expensive equipment not needed; applicable for low volume samples; subtype isolation; medium to high throughput	exosome damage during elution; no universal biomarker; expensive antibodies; problematic elution
PEG	+ + +	+	S—L	+	~ 2 h	easy to use; convenient; several kits available; expensive equipment not needed; medium to high throughput	unstable quality of kits; presence of contaminants
MF	+ + / + + +	+ + / + + +	S	+ + +	~ few h	applicable for low volume samples; continuous process; simultaneous isolation and characterisation; subtype isolation; high throughput	trained personnel needed; low sample capacity; complicated equipment; excessive cost for equipment development

Abbreviations: *S* small volume; *M* medium volume; *L* large volume; *TFF* Tangential Flow Filtration. Other abbreviations are defined in the text

### Comparisons of exosome isolation techniques

Selection of proper exosome isolation method or their combination should be carried out taking into account the following attributes: 1) type of sample; 2) equipment available; 3) yield of exosomes; 4) time of isolation; 5) purity of exosomes; 6) user expertise; 7) volume of sample; 8) if exosomes should be isolated according to subpopulation produced by different cell types. Ideally, exosome isolation method or combination of methods should be rapid, easy to use, effective, reliable, user-friendly, cost affordable, accessible and producing exosomes with high purity and yield. Additionally, the isolation methods should not affect the natural structure of exosomes, nor their biological functions. Advantages and disadvantages of exosome isolation methods specified above are summarised in Table 4.

When the recovery index is taken into account as the main selection criterion, the order of exosome isolation methods is following: PEG-based precipitation (~ 80–90%), UF (~ 60%), DUC (~ 20–40%). The highest purity of exosomes is achieved using DGUC and the lowest one by using PEG-based precipitation. DGUC is the most time and labour-intensive isolation technique, while UF and AC are quite costly. PEG-based precipitation method offers low cost, short time and is less labour-intensive compared to other techniques.

Quality of isolated exosomes needs to be characterised in detail using a battery of sophisticated techniques for characterisation of size and shape of exosomes, i.e. transmission electron microscopy, cryogenic electron microscopy, scanning electron microscopy, atomic force microscopy; size and concentration of exosomes, i.e. nanoparticle tracking analysis and dynamic light scattering; size, shape and charge of exosomes by resistive pulse sensing and tuneable resistive pulse sensing; concentration of exosomes and presence of specific receptors on the exosomal surface by a flow cytometry and fluorescence-based analysis; concentration of exosomes and presence of specific receptors on exosomal surface by surface plasmon resonance; size and shape of exosomes by interferometric reflectance imaging; detection of presence of specific exosomal proteins by Western blotting, ELISA-based assays and mass spectrometry; lipid analyses; next-generation sequencing for DNA analysis, etc. [2, 61, 62]. Single EVs can be studied with several advanced techniques including nano-flow cytometry for multiparametric analysis of surface expressed biomarkers and exosome concentration; super-resolution microscopy of photo-activated localisation microscopy/stochastic optical reconstruction microscopy, quantitative single molecule localization microscopy and super-resolution microscopy for visualisation of small single exosomes; droplet digital ExoELISA for sensitive detection of exosomal cargo; single particle interferometric reflectance imaging for visualisation of exosomes; droplet DNA barcode sequencing for sensitive detection of exosomal DNAs; laser trapping Raman spectroscopy for single molecule analysis of exosomal cargo; hybrid interferometric reflectance imaging–fluorescence microscopy for analysis of protein composition; single-particle spectroscopy for getting spectroscopic (i.e. Fourier Transform Infrared Spectroscopy) fingerprints of individual exosomes and others [1, 38].

## Exosomes as a source of biomarkers

There is a growing evidence that exosomes comprise a new class of biomarkers and this is why they are increasingly investigated as diagnostic tools [63]. In order to have diagnostics, which is robust, specific and at the same time highly accurate it is very important to focus on exosome isolation methods, which are able to enrich subpopulation of exosomes released by a particular tissue in the body. This is a significant challenge since it is estimated that human serum plasma contains 10^10^ EVs and up to 10^16^ lipoproteins per mL of blood plasma [63]. Additionally, biofluids such as human blood plasma/serum contain very high concentration of albumin and other proteins.

Regarding EVs the situation is even more dramatic since it is estimated that blood contain 99.8% of EVs produced by blood cells [64]. Blood cells are able to produce EVs with a steady-state secretion rate of (1.5 ± 0.4) × 10^12^ EVs*/*min [64]. There are significant differences in the ability of cells to produce EVs, for example monocytes are able to produce (45 ± 21) EVs/(min) per cell, while erythrocytes only (3.2 ± 3.0) × 10^–3^ EVs/min per cell. Cell-specific EV secretion rate has not been determined yet in vivo, but results from the cell cultures indicate that the secretion rate can be in the range from ~ 0.08 EVs/min per cell to ~ 1–3 EVs/min and per cell, depending on the type of cells [64]. Thus, plasma/serum EV concentration does not reflect cell abundance in the body and that there are some other factors contributing to EV secretion rate [64]. Furthermore, it was revealed that the exosome secretion rate correlates well with mitochondrial metabolism of producing cells. Conditions leading to generation of reactive oxygen species including several diseases, such as cancers, result in an increased exosome secretion rate and an increased concentration of EVs in plasma/serum [64].

When working with exosomes it is essential to consider a natural adsorption of exosomes on hydrophobic or non-blocked surfaces, this is why it is recommended to work with low-binding Eppendorf tubes or use tubes and other material (like ELISA plates), which are already blocked [38]. Furthermore, it is good to know that a mild agitation might significantly influence integrity of exosomes [38].

Furthermore, identification of promising disease biomarkers can be simplified using artificial intelligence tools such as machine learning algorithms or neural networks especially for high-throughput data analyses [38]. Such approaches can be also used to build models/equations based on analysis of several biomarkers even of different types for “omics” based diagnostics with a more accurate and a robust discrimination power.

From the abovementioned facts, it is clear that in order to use exosomal biomarkers for robust and highly accurate diagnostics of diseases it is very important to focus on exosome isolation methods allowing isolating subpopulation of exosomes produced by a specific type of cells, i.e. cells with cancer. In general we can however state that the technologies to enrich or sort subpopulations of EVs have developed more slowly than characterization tools [1]. One of the most promising methods for such specific exosome isolation is magnetic-based AC method allowing isolation of cell-specific EVs for “liquid biopsy” based diagnostics. Such exosome isolation method can be combined with others like SEC used as a pre-purification step to remove lipoproteins or other types of vesicles [63]. Furthermore, integration of MBs for exosome isolation and their subsequent analysis can be simplified also from a commercial point of view since certain MB types based on the magnetite (Fe_3_O_4_) phase have already been FDA-approved, demonstrating biocompatibility and low toxicity [65]. This is why in the next section we will discuss only approaches for isolation and/or analysis of exosomes based on (bio)affinity interactions using MBs.

## Magnetic AC-based isolation and analysis of exosomes and their content

The main advantages of disadvantages of magnetic AC methods of exosomes isolation are summarised in next Sections. There is additional very strong reason behind use of magnetic AC of exosomes, i.e. the fact that diagnostic industry leaders in the highly automatic machines use magnetic AC capture method for isolation of biomarkers from complex samples. Thus, magnetic AC of exosomes should be quite easily transferrable onto such platforms if strong diagnostic potential of exosomes will be verified. In this section we would like to discuss MB-based AC and/or analysis of exosomes with further division into categories based on (bio)affinity ligand applied for exosome isolation. The section provides a comprehensive overview of magnetic AC based exosome isolation methods and analysis of exosomal content in the last two years (2023–2024) citing ~ 50 references. We provide also a comprehensive Table 5 showing the main characteristics of magnetic AC exosome isolation methods including (bio)affinity ligands used, key analytical/clinical parameters, disease investigated and a source of exosomes. While next Sections describe various approaches either for antibody (Ab)-based isolation of exosomes or aptamer-based isolation of exosomes in details, next Sections also provide lessons learned from such studies with interesting approaches highlighted either for Ab-based AC of exosomes or aptamer-based AC of exosomes.

**Table 5:** Isolation/detection of exosomes using magnetic AC

Biorecognition element	Detection	Linear range (exosomes/mL)	LOD (exosomes/mL)	Disease	Source	Ref
MUC1 aptamer	F	2 × 10^4^—2 × 10^9^	2.9 × 10^3^	GC	MGC-803 cells	[41]
EpCAM & PD-L1 aptamers	SERS	1.1 × 10^6^—1.1 × 10^8^	3.5 × 10^4^	BCa	MCF-7 cells	[42]
EpCAM & PD-L1 aptamers	SERS	*p* < 0.001	AUC = 0.988	BCa	BCa plasma (n = 36)	[42]
CD63 aptamer & CD63 aptamer-QD	F	-	6.5 × 10^4^	leukaemia	HL-60 cells	[86]
CD63 aptamer & nucleolin aptamer-QD	F	*p* < 0.0001	-	leukaemia & other cancers	plasma (n = 50)	[117]
CD63 & MUC1 aptamers	C	1.4 × 10^6^—4.2 × 10^8^	8.9 × 10^5^	alveolar cell carcinoma	A549 cells	[87]
CD63 & MUC1 aptamers	C	*p* = 0.0026	AUC = 0.883	cholangio-carcinoma	serum (n = 18)	[87]
EpCAM, MUC1 & HER2 aptamers	RLS	1 × 10^6^—1 × 10^9^	1.0 × 10^6^	BCa	SK-BR-3 cells	[88]
EpCAM, MUC1 & HER2 aptamers	RLS	*p* < 0.0001	-	BCa	serum (n = 10)	[88]
Imprinted polymer for phospholipids	SERS	-	5.8 × 10^7^	Healthy	urine (n = 1)	[43]
CD63 aptamer	F	8 × 10^7^—1 × 10^10^	4 × 10^7^	human NSCLC	A549 cells	[90]
CD63 aptamer	F	*p* < 0.05	AUC = 0.850	Lung cancer	whole blood (n = 26)	[90]
Anti-EpCAM Ab	E	1 × 10^2^—1 × 10^8^	10	BCa	MCF-7 cells	[111]
Various antibodies	E	*p* < 0.01; *p* < 0.0001	AUC = 0.91–1.00	BCa	plasma (n = 20)	[111]
CD63 & EpCAM aptamers	PEC	1 × 10^5^—1 × 10^11^	2.1 × 10^4^	BCa	MCF-7 cells	[91]
PD-L1 recognised peptide & TiO_2_	SPR	1 × 10^3^—1 × 10^7^	32	BCa	MDA-MB-231 cells	[44]
PD-L1 recognised peptide	SPR	-	AUC = 0.984	BCa	serum (n = 22)	[44]
HER2 Ab	naked eye	1 × 10^8^—1 × 10^9^	8.5 × 10^8^	BCa	HCC1954 cells	[68]
Anti-CD63 Ab	SERS	1 × 10^3^—1 × 10^6^	1 × 10^3^	cancerous exosomes	commercially available exosomes	[71]
EpCAM aptamer	TRL	2.3 × 10^2^ – 2.3 × 10^8^	24	BCa	MCF-7 cells	[92]
EpCAM aptamer	TRL	*p* < 0.0001	-	BCa	plasma (n = 15)	[92]
Anti-EpCAM Ab & anti-EpCAM Ab	F	-	AUC = 0.880	OSCC	saliva (n = 42)	[72]
Anti-EpCAM Ab & anti-CD45 Ab	F	-	AUC = 0.827	OSCC	saliva (n = 42)	[72]
EpCAM aptamer & anti-CD9 Ab	ECL	1 × 10^5^—1 × 10^7^	1.1 × 10^4^	BCa	MCF-7 cells	[93]
CD63 aptamer	MS	-	AUC = 0.924	EC	plasma (n = 105)	[94]
Various aptamers	F, MS, C	Several linear ranges	9.2 × 10^3^ (F) 9.6 × 10^4^ (MS) 6.4 × 10^6^ (UV–Vis)	Bladder cancer	urine (n = 25)	[95]
Various aptamers (CD63, CEA, EpCAM, MUC1, & CA125)	F, MS, C	-	single: AUC = 0.865; multiple: AUC = 0.984	bladder cancer	urine (n = 25)	[95]
Anti-CD63 Ab & anti-PD-L1 Ab	CL	4.8 × 10^3^ – 4.8 × 10^8^	780	LCa	A549 cells	[73]
Anti-CD63 Ab & anti-PD-L1 Ab	CL	-	AUC = 0.939	LCa	serum (n = 52)	[73]
MUC1 aptamer & DC63 aptamer	ECL	1 × 10^4^ – 1 × 10^7^	5 × 10^3^	BCa	MCF-7 cells	[96]
EpCAM aptamer	E	3 × 10^3^ – 5 × 10^5^	280	BCa	MCF-7 cells	[97]
EpCAM aptamer	E	*p* < 0.0001	AUC = 1.0	BCa	plasma (n = 20)	[97]
CD63 aptamer	PT, C, F	Several linear ranges	1.5 × 10^5^ (PT) 2.3 × 10^4^ (C) 5.8 × 10^3^ (F)	GC	SGC-7901 cells	[98]
CD63 aptamer	PT, C, F	p < 0.001	-	GC	serum (n = 20)	[98]
CD63 aptamer	M	7.5 × 10^4^—1.5 × 10^7^	-	BCa	HepG2 cells	[99]
CD63 aptamer	M	*p* ≤ 0.001	-	BCa	serum	[99]
CD63 aptamer	F	1 × 10^5^ – 1 × 10^10^	4.2 × 10^4^	BCa	HepG2 cells	[100]
Phospholipid isolation	MS	-	AUC = 0.980 −1.000	AD	serum (n = 238)	[45]
Peptide C3	C	Several linear ranges	3.3 – 4.7 × 10^5^	BCa	MCF-7, MDA-MB231 and SKBr3 cells	[46]
Peptide C3	E	Several linear ranges	1.7 – 2.6 × 10^5^	BCa	MCF-7, MDA-MB231 and SKBr3 cells	[46]
CD63 aptamer & EpCAM aptamer	E	1 × 10^5^—1 × 10^10^	4.3 × 10^4^	BCa	4T1 cells	[101]
PD-L1 aptamer	TRL	1.1 × 10^5^—1.1 × 10^10^	190	melanoma	B16-F10 cells	[102]
PD-L1 aptamer	TRL	*p* < 0.0001	-	LCa	whole blood	[102]
anti-EphA2 Ab & anti-glypican-1 Ab	MS	-	AUC = 1.000 (ELISA 1 AUC = 0.988 ELISA 2 AUC 0.902)	PDAC	serum (n = 43)	[77]
anti-EpCAM Ab &anti-glypican-1 Ab	FET	-	AUC = 0.980	PDAC	serum (n = 20)	[78]
Anti-CD63 Ab & PD-L1 aptamer	F	500—5 × 10^4^	100	nasopharyngeal carcinoma	C666-1 cells	[80]
Anti-CD63 Ab & PD-L1 aptamer	F	*p* < 0.0001	-	nasopharyngeal carcinoma	plasma (n = 20)	[80]
TiO_2_ phospholipid & anti-glypican-1 Ab	MS	7.1 × 10^4^—7.1 × 10^9^	-	PDAC	PANC-1 cells	[112]
TiO_2_ phospholipid & anti-glypican-1 Ab	MS	*p* < 0.0001	AUC = 0.957	PDAC	serum (n = 45)	[112]
Anti-CD63 Ab	CL	3.1 × 10^3^—3.1 × 10^8^	2.1 × 10^3^	LCa	A549 cells	[81]
Anti-CD63 Ab	CL	*p* < 0.0001	-	Various cancers	serum (n = 54)	[81]
EpCAM aptamer	E	2.2 × 10^4^ – 2.2 × 10^8^	2.2 × 10^4^	BCa	SK-BR-3 cells	[103]
EpCAM aptamer	E	-	-	BCa	serum (n = 20)	[103]
Anti-CD9 Ab & anti-EpCAM Ab	SERS	1.5 × 10^8^ – 7.4 × 10^9^	1.5 × 10^8^	OCa	OVCAR3 cells	[82]
PD-L1 aptamer	F	2.9 × 10^6^ – 2.9 × 10^10^	1.7 × 10^6^	NSCLC	NCI-H1975 cells	[108]
PD-L1 aptamer	F	*p* < 0.001	AUC = 0.89 (ELISA AUC = 0.76)	NSCLC	plasma (n = 30)	[108]
Phospholipid detection & CD63 aptamer	C	4.3 × 10^5^ – 4.3 × 10^8^	8.6 × 10^4^	BCa	MCF-7 cells	[104]
CD63 aptamer & nucleolin aptamer	C	1 × 10^5^ – 1 × 10^6^	4.5 × 10^4^	leukaemia	HL-60 cells	[105]
Anti-annexin V Ab & Anti-EGFR Ab	F	2.5 × 10^6^ – 1 × 10^8^	3.7 × 10^5^	OSCC	A549 cells	[83]
Anti-annexin V Ab & Anti-EGFR Ab	F	*p* < 0.0001	-	OSCC	saliva (n = 85)	[83]
CD63 aptamer	F	-	AUC = 0.725	LCa	plasma (n = 48)	[106]
PMSA aptamer	SERS	1.2 × 10^5^ – 2.4 × 10^6^	1.9 × 10^4^	PCa	LNCaP cells	[107]
PMSA aptamer	SERS	*p* = 0.0041	-	PCa	serum (n = 19)	[107]

Abbreviations: *E* electrochemical; *F* fluorescent; *GC* gastric cancer; *BCa* breast cancer; *C* colorimetric; *RLS* resonance light scattering; *PEC* photoelectrochemical; *SPR* surface plasmon resonance; *TRL* time-resolved luminescence; *OSCC* oral squamous cell carcinoma; *EC* endometrial cancer; *CL* Chemiluminescnece, *GC* gastric cancer; *PT* photothermal; *M* magnetic; *AD* Alzheimer disease; *EphA*2 ephrin type A receptor 2; *PDAC* pancreatic dual adenocarcinoma; *FET* field-effect transistor; *LCa*: lung cancer; *OCa* ovarian cancer; *NSCLC* non-small cell lung cancer

### Antibody (Ab)-based AC of exosomes

Magnetic-based immunoassay was applied for isolation of exosomes from psoriasis induced cell line HaCaT [66]. Proteomic analysis using MS applied to identify proteins expressed in induced and uninduced/control cell lines revealed 2,796 protein, from which 20 proteins were differentially expressed in induced cell line compared to the uninduced/control cell line with a potential to use them as potential biomarkers [66].

MBs modified by Ti layer and cyclodextrin molecules were used for host–guest based immobilisation of anti-CD63 antibody [67]. Interaction of such a hybrid MBs with exosomes was strong and attributed to two types of interactions, i.e. via interaction of Ti layer with phospholipids of exosomes and via bioaffinity capture of exosomes using anti-CD63 antibodies. Furthermore, a mild elution procedure was able to release exosomes for further LC–MS analysis of proteins. The method of exosome isolation was more effective compared to UC with twofold increased amount of proteins captured and the MB-isolation method allowed to identify 1,060 unique exosomal proteins compared to UC-based isolation method, which identified 768 unique exosomal proteins [67].

**Fig. 5:**
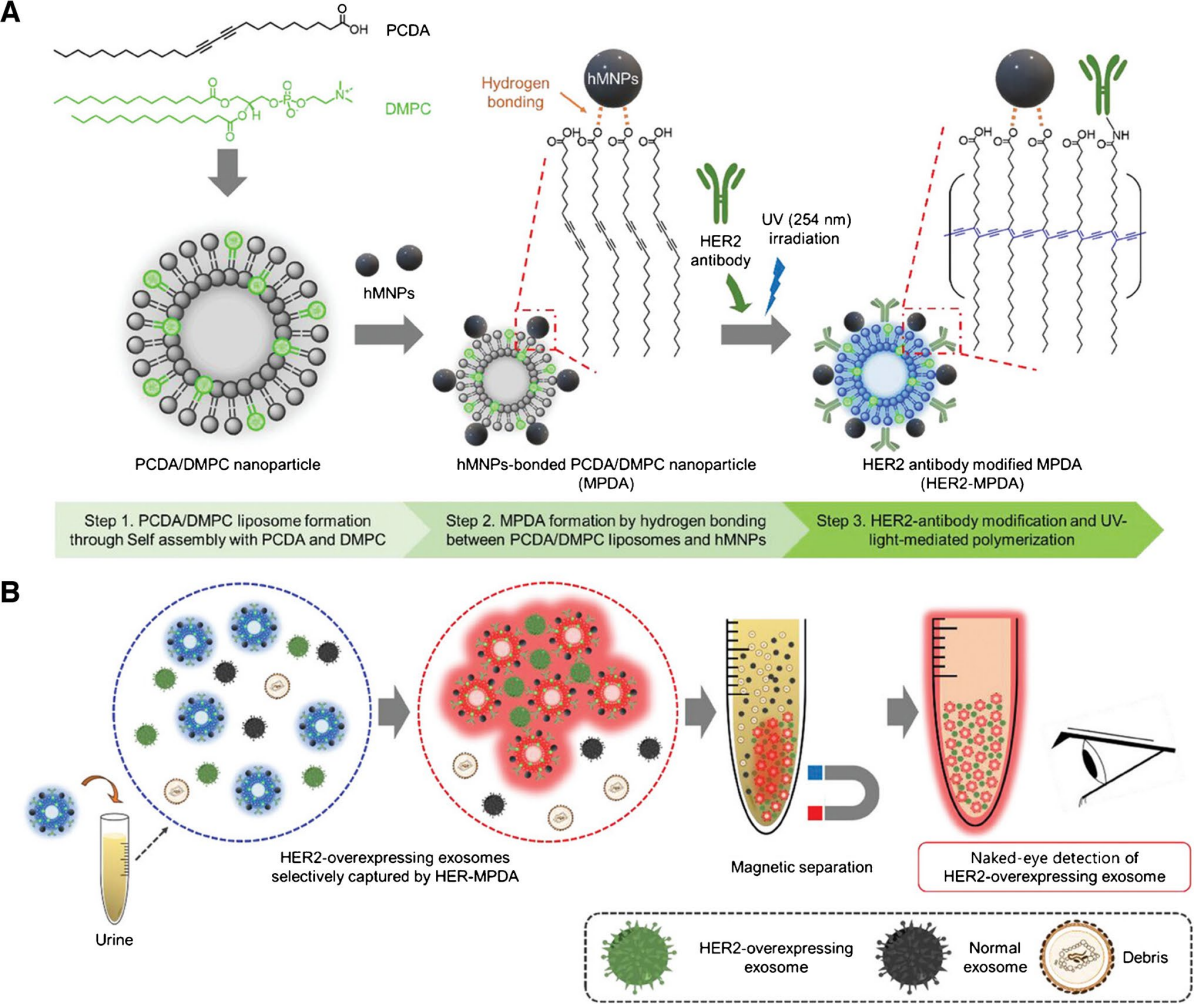
Colorimetric detection of HER2-overexpressing-cancer-derived exosomes in urine using magnetic-responsive polydiacetylenes (PDA) nanoparticles (NPs). (**a**) Schematic procedure of HER2-MPDA synthesis, a colorimetric sensor for detect HER2-overexpressing exosomes: Step 1: Synthesis of PCDA/DMPC NPs by thin-film hydration method using PCDA and DMPC. Step 2: Formation of MPDA by hydrogen bonding with PCDA/DMPC NPs and hydrophilic magnetic NPs (hMNPs). Step 3: Fabrication of HER2-MPDA by conjugating HER2 antibody on MPDA and photopolymerization using UV irradiation. (**b**) Schematic illustration of colorimetric detection of HER2-overexpressing exosomes in non-purified urine samples using HER2-MPDA. Reproduced with permission from ref. [68]. Copyright (2024) John Wiley and Sons

A low-cost, equipment-free, and easy-to-use polydiacetylene (PDA)-based colorimetric sensor for isolation and detection of exosomes was developed (Fig. 5) [68]. MBs containing a lipid layer forming a liposome with attached HER2 antibodies were used for isolation of exosomes. The liposome contained functional groups, which upon irradiation changed the colour from blue to red and thus presence of isolated exosomes could be confirmed by a naked eye. Two cell lines were used in the study, i.e. HCC1954 (HER2 +) and HCC1143 (HER2-) with LOD down to 8.5 × 10^8^ exosomes/mL [68].

**Fig. 6:**
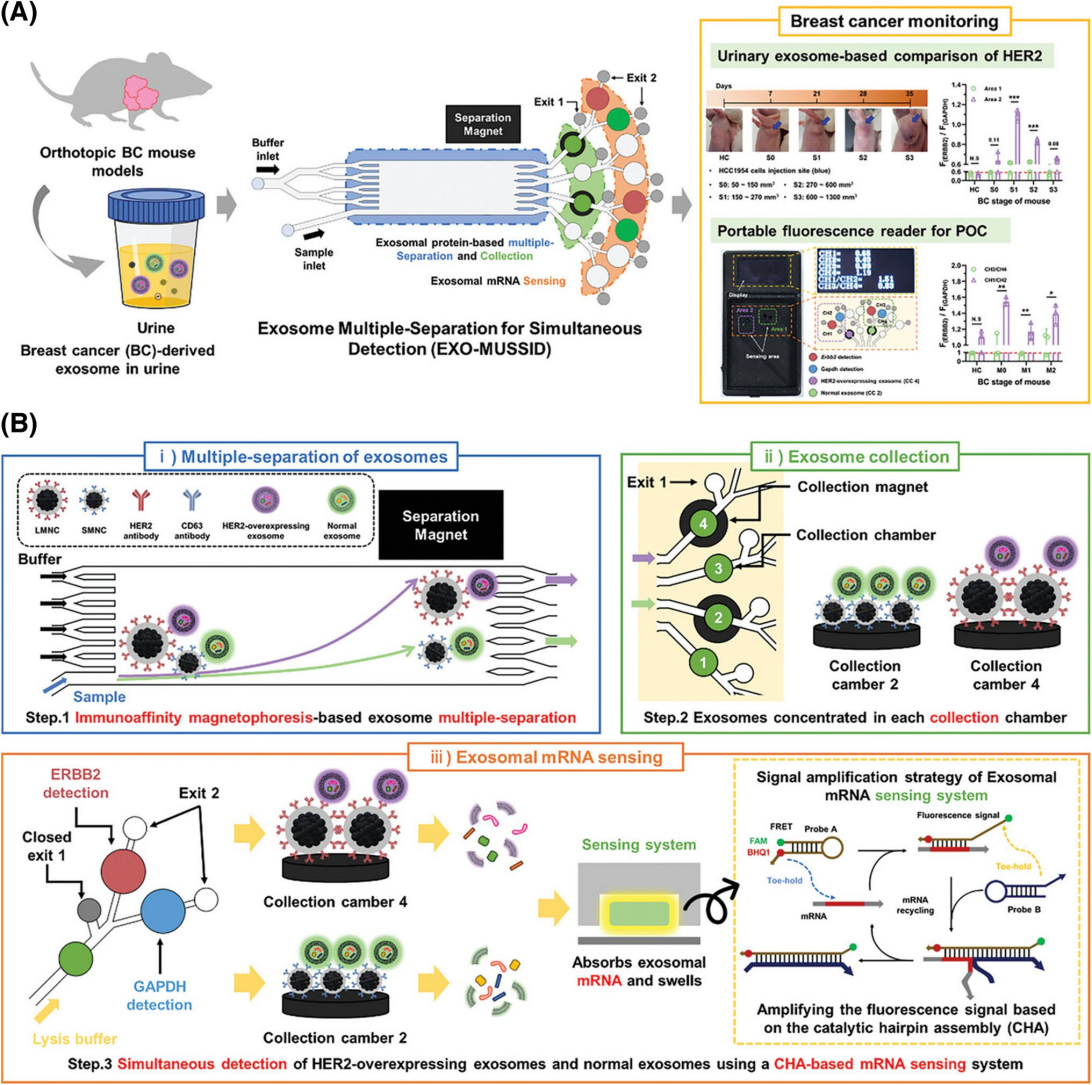
Schematic illustration of the overall process of the EXO-MUSSID platform. (**a**) The EXO-MUSSID platform is a monitoring system to evaluate the therapeutic efficacy of HER2-overexpressing breast cancer (HER2+ BC) through one-step detection of exosomal proteins and mRNA in urine samples. The additionally developed portable fluorescence reader can be used for point-of-care (POC) monitoring of HER2 (ERBB2) expression changes. (**b**) The detailed study design is as follows: i) Separation of HER2-overexpressing exosomes and normal exosomes using MNCs (HER2-MNC and CD63-MNC) with different magnetization properties; ii) Separation of magnetically labeled exosomes based on differences in flow velocity and magnetization; exosomes are collected and concentrated in a collection chamber; iii) Injection of lysis buffer to elute the mRNA in the exosomes, and simultaneous detection of the target *Erbb2* (red) and *Gapdh* (blue) in the sensing gel carrying the target recycling fluorescence amplification system. All processes are performed on one platform. Reproduced from an open access publication, from ref. [69]

A point of care device with a portable fluorescence reader was applied for isolation of exosomes and for subsequent monitoring of the therapeutic efficacy of drugs (Fig. 6) [69]. Different types of exosomes were isolated at the same time using two different types of MBs with a larger size (407 nm) and a smaller size (224 nm). Smaller MBs were modified by anti-CD63 antibodies, while larger MBs by anti-HER2 antibodies. The device separated both types of MBs in different channels using their different magnetisation properties and finally those two types of MBs ended up in different chambers. Finally, mRNA analysis was performed in both chambers. Exosomes were isolated from two different cell lines HCC1954 and HCC1143 with the efficiency of 91.3%. Analysis of HER2 expression in exosomes isolated from different culture medium showed ~ (27.9 ± 0.21)-fold higher expression in HCC1954 exosomes compared to HCC1143 exosomes. The device was then applied for exosome isolation and exosomal mRNA analysis in mouse urine depending on BCa stage and also for monitoring of efficacy of drug treatment [69]. A similar approach from the same group was also used for isolation and detection of BCa exosomes produced by a HCC1954 cell line (HER2-positive) [70].

MBs modified by a layer of mesoporous silica coating were used for growth of Au nanorods (AuNR) within the pores of mesoporous silica [71]. Finally, AuNPs were grown on AuNR and to such hybrid MBs antiCD63 antibodies were immobilised for isolation of exosomes. Two types of commercially available exosomes (normal and cancerous) were applied in the study and analysed using SERS. The SERS spectra were treated using principal component analysis (PCA). The device was able to detect exosomes with LOD of 1 × 10^3^ exosomes/mL [71].

A microfluidic microchip was used for magnetic isolation of two different types of exosomes (i.e. produced by tumour or immune cells) from saliva [72]. MBs were modified by anti-PD-L1 antibodies and for specific detection of cancerous cells fluorescent nanoprobe NP1 targeting EpCAM (cancerous exosomes) and fluorescent nanoprobe NP2 targeting CD45 (immune exosomes) were used in the system. The device was applied for isolation of exosomes from Cal-27 cell line (a human oral cancer cell line) and Raji cells (an immune cell line). Finally, analysis of saliva specimens from 37 oral squamous cell carcinoma patients and 5 HC was performed showing excellent discrimination power when using count of PD-L1 positive exosomes produced by tumour cells (AUC = 0.880) and using of PD-L1 positive exosomes produced by immune cells (AUC = 0.827) [72].

MBs modified by anti-CD63 antibody were applied for exosomes isolation and exosome detection was performed using a detection probe composed of a hybrid nanoparticle modified by anti-PD-L1 antibody exhibiting a strong chemiluminescent signal [73]. The device was able to detect exosomes produced by A549 cell line with LOD of 7.8 × 10^2^ exosomes/mL. The recovery of exosome analysis was from 88.4 to 100.0%. A clinical performance of the device was evaluated by analysis of exosomes isolated from serum of 20 HC and 32 LCa patients (13 with minimally invasive carcinoma and 19 with invasive carcinoma). The device was able to discriminate HC from LCa patients with AUC of 0.939 and patients with minimally invasive LCa from patients with invasive LCa with AUC of 0.753 [73].

**Fig. 7:**
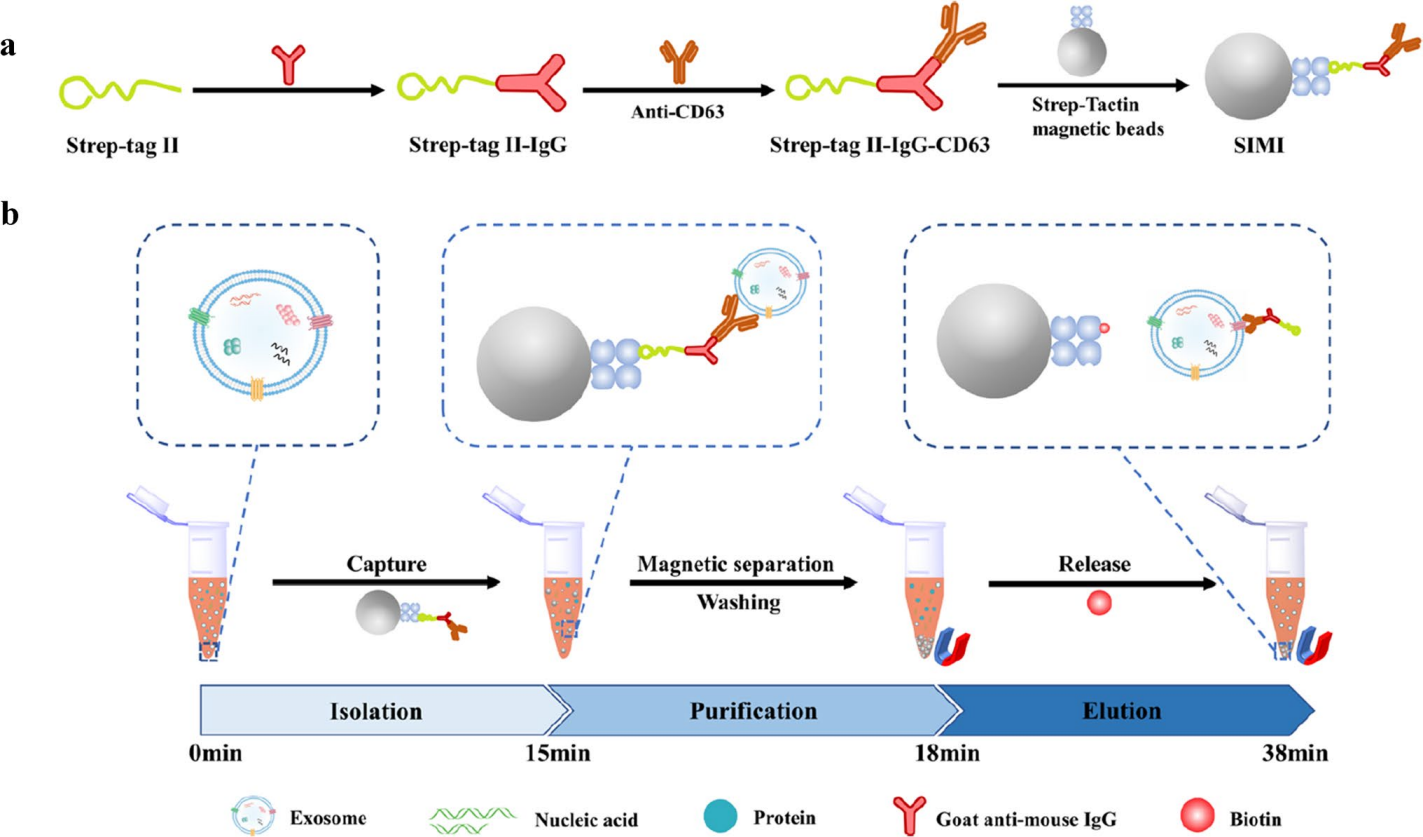
Schematic diagram of the SIMI-based isolation of exosomes. (**a**) Preparation procedure of SIMI magnetic beads and (**b**) exosome isolation process of the SIMI system. Reprinted with permission from ref. [75]. Copyright (2024) American Chemical Society

A MB-based microfluidic platform was applied for isolation of highly pure exosomes using rapid isolation procedure (29 min) (Fig. 7) [74]. The system had integrated bubble-based micromixer and was easy to fabricate and operate. The system could isolate exosomes with 75.8% isolation efficiency and 62.7% release efficiency. The exosome release system was based on the use of Strep-Tactin modified MBs to which antibodies were immobilised for immunocapture of exosomes via Strep-tag II. Exosomes were released from MBs using biotin. The system was applied for analysis of human plasma samples from 4 HC and 4 hepatocellular carcinoma (HCC) patients with a significant discrimination power represented by p < 0.01 [74]. A similar approach from the same group was applied for isolation of exosomes with efficiency of 82.5% and a release efficiency of 62% and the isolation was completed within 38 min, when working in the Eppendorf tube. The isolation system was much more effective compared to UC isolation of exosomes exhibiting isolation efficiency of only 19.7% [75].

Anti-CD63 antibodies immobilised onto MBs together with a redox probe were used for isolation of exosomes produced by A549 cell line and subsequently for electrochemical analysis of exosomes [76].

MBs were modified by anti-EphA2 (ephrin type A receptor 2) antibody for isolation of exosomes produced by pancreatic cell line PANC-1 [77]. Detection of pancreatic exosomes was accomplished by incubation of MBs with attached exosomes with AuNPs modified by anti-glypican-1 antibody. MS was then used to detect AuNPs and the assay could detect as low as 78 pg/mL of glypican-1 protein. The assay protocol was applied for analysis of serum samples from 22 HC and 21 pancreatic cancer (PaCa) patients (early stage I/II). The assay showed a very good discrimination power represented by AUC of 1.000, while an ELISA based on glypican-1 protein analysis showing AUC of 0.988 and ELISA based on analysis of CA 19–9 protein detection exhibiting AUC of 0.902 [77].

MBs modified by two antibodies, i.e. anti-EpCAM and anti-glypican-1 antibodies were used for selective isolation of exosomes produced by pancreatic cancer cell line PANC-1 [78]. Exosomes were lysed and miRNA10b released was measured using field-effect transistor (FET) sensing using a peptide nucleotide probe for a selective capture of miRNA10b. The assay was then applied for analysis of serum samples from 10 HC and 10 PaCa patients with AUC of 0.980, while CA19-9 (carbohydrate antigen) offered AUC of 0.790 and CEA (carcinoembryonic antigen) offered AUC of 0.760 [78].

Yu et al. identified conditions for continuous isolation of stem-cell derived exosomes by recycled MBs with immobilised anti-CD63 antibodies [79]. The study identified MgCl_2_ as the best elution solution compared to NaOH or glycine–HCl buffer [79].

Anti-CD63 antibody modified MBs were used for isolation of exosomes from nasopharyngeal carcinoma cell line C666-1 [80]. Then PD-L1 aptamer was added to exosomes bound to MBs and DNA sequence of the aptamer was elongated in presence of transferase. Elongated DNA sequence was then recognised by Cas12a and crRNA, which was able to cleave DNA labelled by a fluorescent dye and a quencher releasing a fluorescent dye with signal on fluorescent signal generated. Exosomes were detected with LOD of 100 exosomes/mL [80].

A hybrid type of nanoparticles based on MBs onto which AuNPs, Ag shell and complex of Co^2+^ were deposited was used as a probe exhibiting strong and stable chemiluminescent signal [81]. Isolation of exosomes produced by human lung cancer cell line A549 was possible by immobilisation of anti-CD63 antibodies onto such hybrid particle and an ECL signal was generated in presence of hydrogen peroxide. The sensor detected exosomes with LOD down to 2.1 × 10^3^ exosomes/mL. Finally, the approach was applied for analysis of human serum samples from 21 HC, 19 LCa patients, 10 BCa patients and 4 liver cancer patients and the sensor was able to discriminate HC from patients of all cancer types with *p* < 0.0001 [81]. This suggests that CD63 as a receptor is a general cancer marker and cannot be used for diagnostics of specific types of cancer.

**Fig. 8:**
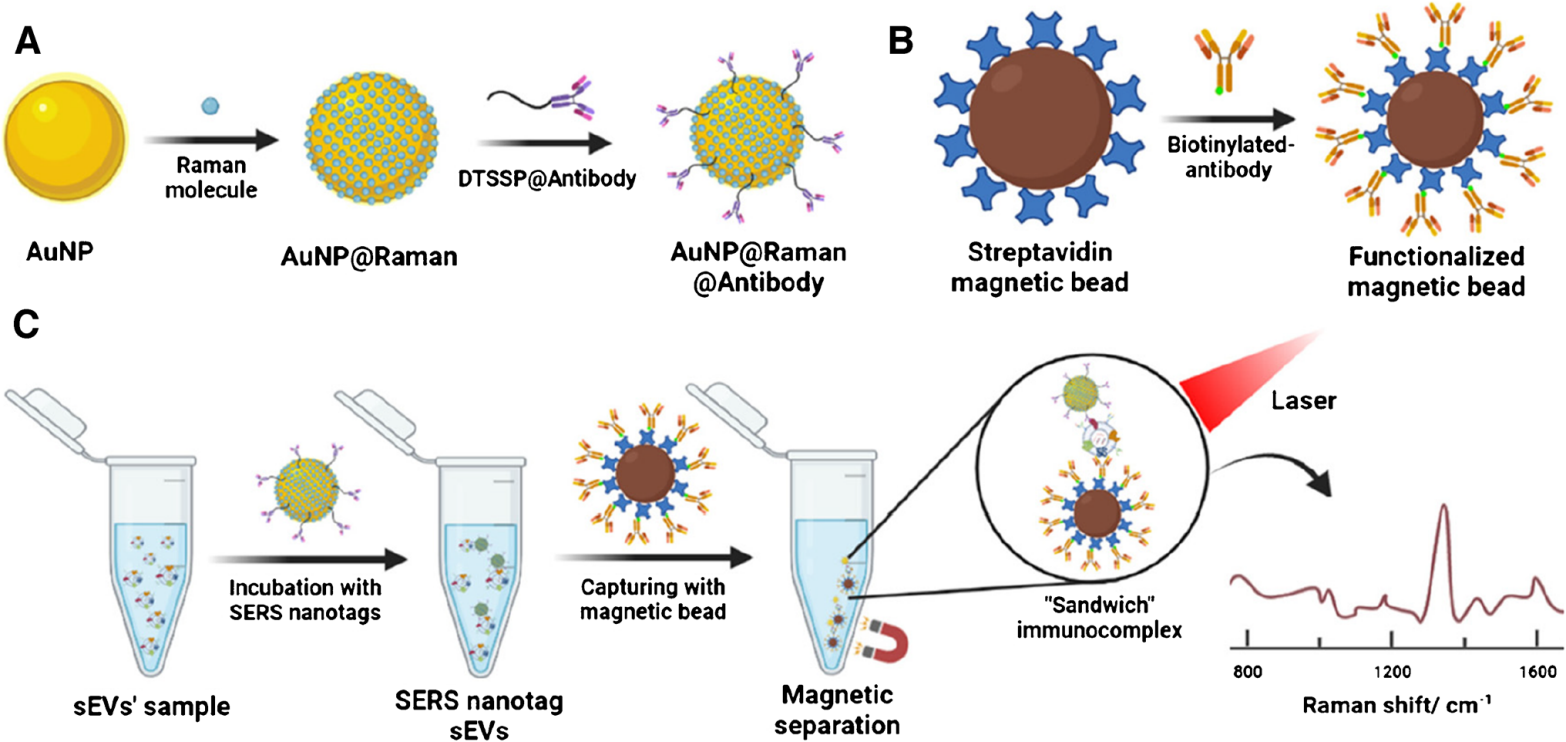
Schematic of the “sandwich” SERS immunoassay. (**a**) Preparation of SERS nanotags including coating the gold nanoparticles (AuNP) with Raman molecules, then conjugating with antibodies which bind to the corresponding proteins of sEV surface. (**b**) Functionalization of magnetic beads with anti-tetraspanin antibodies to capture sEVs from ovarian cancer cell lines. (**c**) Forming and detecting of “sandwich” immunoassay of sEVs by Raman spectroscopy. Reproduced with permission from ref. [82]. Copyright (2023) Royal Society of Chemistry

MBs conjugated with anti-CD9, anti-CD63 or anti-CD81 antibodies were tested for isolation of exosomes, which were detected using a SERS probe composed of AuNPs modified by a SERS tag and one of three antibodies, i.e. anti-EpCAM, anti-CA125 or anti-CD24 antibodies (Fig. 8) [82]. Exosomes produced by ovarian cancer cell line OVCAR3 could be detected with LOD of 1.5 × 10^8^ exosomes/mL [82].

Exosomes produced by oral squamous cell carcinoma were isolated using MBs modified by anti-annexin V or anti-EGFR antibodies and detection of exosomes was accomplished using biotinylated anti-EGFR antibodies to which streptavidin-QDs conjugate was attached for a fluorescent signal reading [83]. Annexin V positive exosomes were detected with LOD of 3.7 × 10^5^ exosomes/mL, while annexin V negative exosomes with LOD of 6 × 10^5^ exosomes/mL. When analysis of saliva samples from OSCC patients (70) were compared to HC (10), a nice discrimination power was obtained with *p* < 0.0001 with a potential to discriminate also different cancer stages [83].

Commercially available MB-based exosome kit was applied for isolation of exosomes from urine [84]. The results indicate that exosomes in urine were stable, when urine was stored for 6 h at room temperature or for 12 h at 4 °C, but there were significant variations in exosome amount, when urine was collected from the same individual at different time points, especially under different diet conditions. The study also revealed that the results should be normalised to creatinine content in urine. Exosomes isolated were lysed and analysed using liquid chromatography combined with mass spectrometry (LC–MS). The analysis revealed presence of 47,334 unique peptides representing 4,939 proteins. Five proteins, i.e. AKAP4, TRIM28, FGFR3, MMP11 and ATP5PB were identified as the most promising to discriminate prostate cancer (PCa) patients from heathy individuals with AUC ranging from 0.68 to 0.78. When all 5 proteins were combined into the panel, discrimination power increased to AUC of 0.988 [84].

### Lessons learned from antibody-based AC of exosomes

We can conclude that there are different applications of antibody-based AC either for quantification of exosomes or for isolation of exosomes with a subsequent analysis of exosomal cargo.

Quantification of exosomes can be done using only one antibody attached to MBs with some tag already attached to MBs with the final quantification of exosomes either by a naked eye (a colorimetric signal) [68], reading a SERS signal [71], an electrochemical signal [76] or a chemiluminescent signal [81]. There is also an option that isolation of exosomes is done using primary antibody (Ab_1_) modified MBs and the signal is generated using a second antibody (Ab_2_) attached to a nanoprobe being a tag or bearing a tag with the final quantification of exosomes performed by reading of fluorescence [72, 80, 83], chemiluminescence [73], MS spectra [77] and SERS signal [82].

There are also studies showing that exosome isolation was performed by MBs modified by Ab_1_ and then two different antibodies attached to fluorescent NPs (i.e. Ab_2_ to NP_1_ or Ab_3_ to NP_2_) were used to divide population of exosomes into sub-populations (i.e. tumour exosomes or immune exosomes) with subsequent quantification of immune or tumour exosomes reading fluorescence [72]. A similar approach was used for isolation of exosomes using MBs modified by Ab_1_ with clustering of exosomes into three distinct subpopulations based on expression of exosomal receptors using three different Abs conjugated to SERS probes (i.e. Ab_2_ to SERS probe_1_; Ab_3_ to SERS probe_2_, Ab_4_ to SERS probe_3_) [82].

One approach described use of two different bioaffinity capture ligands, i.e. isolation of exosomes by Ab modified MBs with a fluorescent signal generation using a fluorescent aptamer probe [80]. Interestingly a hybrid capture protocol relaying on simultaneous isolation of exosomes by MBs with immobilised Abs and with attached Ti^4+^ cations (interacting with phospholipids) was applied for exosome isolation [67].

There are also several different protocols applied for analysis of exosomal cargo after exosomes were isolated by magnetic AC approach. The simplest way was a direct lysis of exosomes still present on MBs [78]. There are also protocols describing release of exosomes isolated by AC approach using different release agents including ammonia [67]; MgCl_2_, NaOH or glycine–HCl buffer [79].

The most sophisticated release protocol applied was based on modification of MBs by strep-tactin with a moderate affinity towards Strep II tag [74, 75]. Abs were covalently linked to Strep II tag and attached to strep-tactin modified MBs via moderate affinity interactions. Such MBs were then incubated with exosomes and finally exosomes were released from MBs using biotin disrupting moderate affinity interaction between Strep II tag and strep-tactin [74, 75].

Finally, an approach using MBs with two different sizes, which were modified by two different antibodies (i.e. MBs_1_ with Ab_1_ and MBs_2_ with Ab_2_) was developed [69, 70]. Different subpopulation of exosomes bearing different receptors (recognised by either by Ab_1_ or Ab_2_) were separated within a microfluidic device based on the size of MBs with a subsequent lysis of exosomes still attached to MBs. The separation of a lysate from MBs was performed using the device with a fluorescent analysis of mRNA performed in a separate chamber (free of any MBs) [69, 70].

### Aptamer-based AC of exosomes

DNA walker triggered detection of exosomes was accomplished using MBs modified by fluorescent substrate strands and motor DNA strands [41]. MUC1 aptamer was hybridised on a DNA motor and upon interaction with exosomes produced by gastric cancer cell (GC) line MGC-803, MUC1 aptamer was detached from the DNA motor, which became active and in presence of restriction nuclease, DNA motor starts to walk autonomously along the MB surface cleaving substrate strands with turn on fluorescent signal obtained. The fluorescence intensity can monitor the process in real-time. The method was able to detect exosomes with LOD of 2.9 × 10^3^ particles/mL, demonstrating excellent selectivity [41].

Su et al. developed an approach for specific detection of programmed cell death ligand 1 (PD-L1) positive tumour derived exosomes [42]. The assay included MBs modified by EpCAM aptamer and a polymer for quick (30 min) capture of exosome with a high efficiency of 90.5% and specificity. In the next step, PD-L1 positive exosomes were recognized and quantified using PD-L1 aptamer and the SERS signal was generated in presence of substrate and aptamer-functionalised gold nanoprobe. The method was able to detect exosomes from BCa cell line MCF-7 with LOD of 3.5 × 10^4^ exosomes/mL and even was able to discriminate plasma samples from BCa patients from healthy control with AUC of 0.988 [42].

MBs were modified by DNA molecules onto which a rolling circle amplification (RCA) strategy secured in situ grow of multivalent DNA aptamers recognising CD63 [85]. Near infrared light irradiation destroyed the secondary structure of aptamer releasing exosomes, which could be then used for other types of assays [85].

An approach allowing detecting several analytes present on exosomes was based on integration of fluorescent DNA assemblies made of quantum dots (QDs) and aptamers using a RCA strategy [86]. Exosomes were enriched using MBs modified by CD63 aptamer. The signal was generated using a fluorescent DNA assembly made of CD63 aptamer or nucleosin aptamer. The method was able to detect exosome produced by leukaemia cell line HL-60 down to 6.5 × 10^4^ exosome/mL. Moreover, the method was also applied for analysis of 50 serum samples (20 HC and from patients with cancer affecting the following organs: 10 leukaemia, 6 lung, 5 liver, 5 breast and 4 thyroid) using both aptamers. All cancerous patients could be discriminated from healthy individuals with *p* < 0.0001 [86].

MBs modified by CD63 aptamer were used for exosome isolation with subsequent detection of exosomes using other nanoprobe based on AuNPs loaded with DNA primer and MUC1 aptamer. The signal was amplified using DNA transferase, which made biotinylated DNA sequences. The next step was incubation with streptavidin-HRP conjugate delivering multiple HRP molecules to exosomes for colorimetric detection. Alveolar cell carcinoma cell line A549 was detected with LOD of 8.9 × 10^5^ exosomes/mL. Furthermore, the approach was tested by analysis of serum samples from HC (7) and cholangiocarcinoma patients (11) with a significant discrimination (AUC = 0.883) [87].

A dual mode assay allowed to detect exosomes either using flow cytometry or resonance light scattering [88]. MBs were modified by EpCAM aptamer and in the system two DNA probes, i.e. containing MUC1 aptamer or HER2 aptamer were applied. Presence of both receptors on the surface of exosomes recognised by both DNA probes allowed proximity ligation mediated RCA reaction. Addition of complementary scattering DNA probe made of AuNPs allowed to read two different signals. Exosomes produced by BCa cell line SK-BR-3 could be detected with LOD of 1.0 × 10^6^ exosomes/mL. Analysis of exosomes from human serum samples showed a better discrimination between BCa patients (*n* = 5) and HC (*n* = 5) for resonance light scattering (*p* < 0.0001) than for flow cytometry (*p* < 0.001) [88].

MBs covered by MoS_2_ nanomaterial, AuNPs and EpCAM aptamers were applied for isolation of exosomes from human plasma of BCa patients [89]. When the isolation of exosomes was completed, exosomes were released from modified MBs by a complementary strand. In the next step exosomes were purified from complementary DNA strands using SEC. NTA confirmed that 1.0 × 10^7^—1.0 × 10^8^ exosomes were isolated from 1 mL of plasma of BCa patients. Finally, the interaction of exosomes with epithelial cells was investigated [89].

A ratiometric fluorescent approach based on MBs with Cy5-labelled CD63 aptamer and another fluorescent dye was proposed for isolation and label-free detection of exosomes [90]. Upon interaction of modified MBs with exosomes the second fluorescent dye was released signalling presence of exosomes on MBs. The approach offered LOD of 4 × 10^7^ exosomes/mL, when using exosomes produced by human non-small cell lung cancer cell line A549. The method offered recovery index from 100 to 111% with RSD from 10.0 to 18.4%. The assay was also applied for analysis in whole blood of LCa patients and HC with AUC = 0.850 [90].

MBs modified by CD63 aptamer were used for isolation of exosomes [91]. In the next step EpCAM aptamers were applied for interaction with EpCAM exosomal receptors. In presence of nucleotide transferase, EpCAM aptamer was enlarged and then DNA sequence containing alkaline phosphatase (ALP) was added to have multiple ALP molecules along enlarged EpCAM aptamer. Finally, MBs with captured exosomes and ALP molecules attached to exosomes were deposited on the electrode surface and photoelectrochemical signal was read. Exosomes produced by MCF-7 cell line could be detected with LOD of 2.1 × 10^4^ exosomes/mL and the recovery of analysis was in the range 98%−104% [91].

**Fig. 9:**
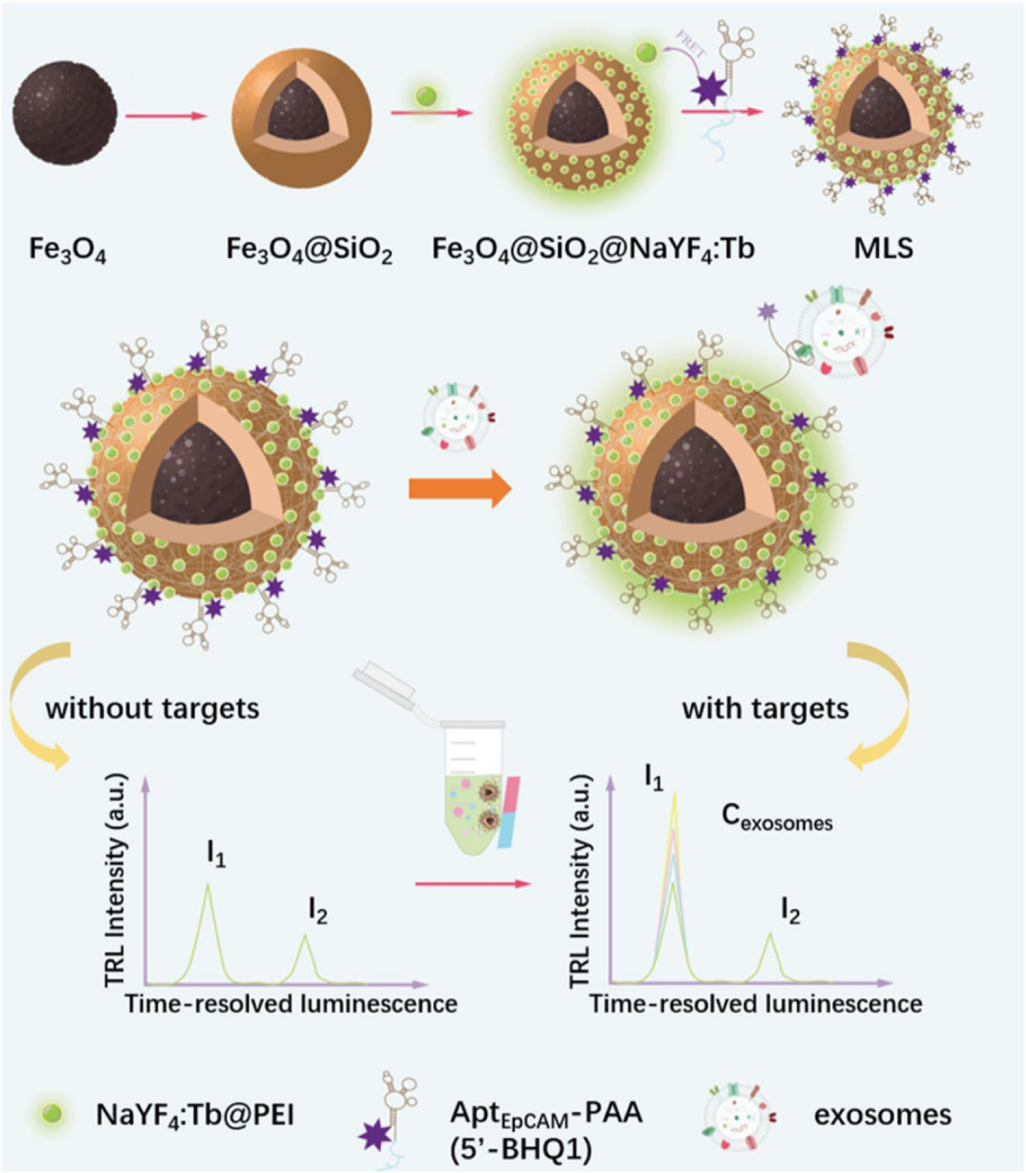
Illustration of the working principle for high-sensitive detection of exosomal EpCAM based on MLS. Reprinted with permission from ref. [92]. Copyright (2024) Royal Society of Chemistry

A magnetic lanthanide sensor (MLS) was developed for sensitive detection of exosomes based on FRET strategy (Fig. 9) [92]. MBs were modified by SiO_2_ layer to which terbium-doped NaYF nanoparticles, deformable EpCAM aptamers terminated by a BHQ1 quencher were immobilised. Without any exosome present on the modified MB surface there was low time-resolved luminescence signal, while upon attachment of exosomes, the aptamer changes its conformation and the BHQ1 quencher was moved away from the surface resolving the signal. Exosomes produced by MCF-7 cells were detected with LOD of 24 exosomes/mL. Furthermore the device was applied for analysis of exosomes isolated from plasma samples of 5 HC, 5 BCa patients and 5 lung cancer (LCa) patients. Two cancerous samples (BCa *vs.* LCa) could be detected with *p* = 0.0041 and both cancerous samples can be discriminated from HC with *p* < 0.0001 [92].

MBs modified by peptide and one of several aptamers (EpCAM, CEA, AFP, CD63, PD-L1, HER2 and MUC1) were used for isolation of exosomes [93]. The signal was generated using a signal probe composed of silica NPs loaded with an ECL reagent and an anti-CD9 antibody for capture of exosomes and for generation of a ECL signal. Exosomes produced by MCF-7 were analysed with LOD of 1.3 × 10^4^ exosomes/mL. The device with seven different MBs was then used to isolate exosomes produce by 7 different cell lines, i.e. 5 cancerous cell lines (MDA-MB-231, HeLa, A549, HepG2, and MCF-7) and two healthy cell lines (MCF-10A and 293 cells). Exosomes produced by different cell lines expressed different pattern of exosomal proteins and it can be concluded that the expression of seven receptors were upregulated in exosomes produced by cancerous cell lines compared to the “healthy” exosomes [93].

MB modified by AuNPs and CD63 aptamer were used for isolation of exosomes [94]. In the subsequent step, such MBs with exosomes attached were deposited on a MALDI-TOF plate for MS analysis. Data obtained were then treated using support vector machine algorithms and used for analysis of samples from 51 endometrial cancer patients and 54 benign controls with an excellent AUC = 0.924 obtained [94].

**Fig. 10:**
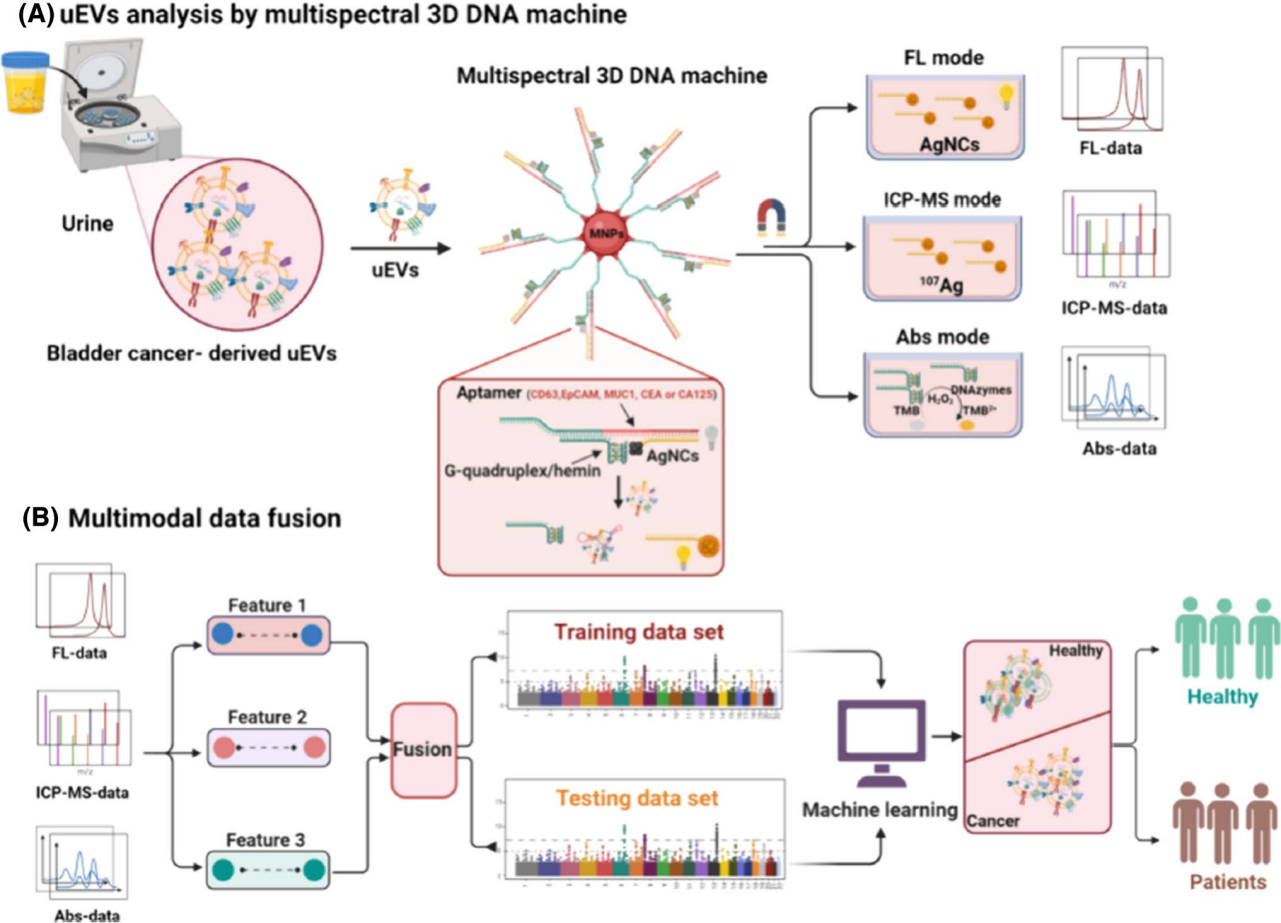
Schematic Illustration of the Multispectral 3D DNA machine in combination with machine learning for noninvasive and precise diagnosis of BCa cancer: (**a**) the collection of multimodal data of uEVs from naturally voided urine by the 3D DNA Machine; (**b**) an overview of multimodal ML algorithm-based uEVs classification using uEVs multimodal signal patterns*.* reproduced with permission from ref. [95]. Copyright (2024) American Chemical Society

**Fig. 11:**
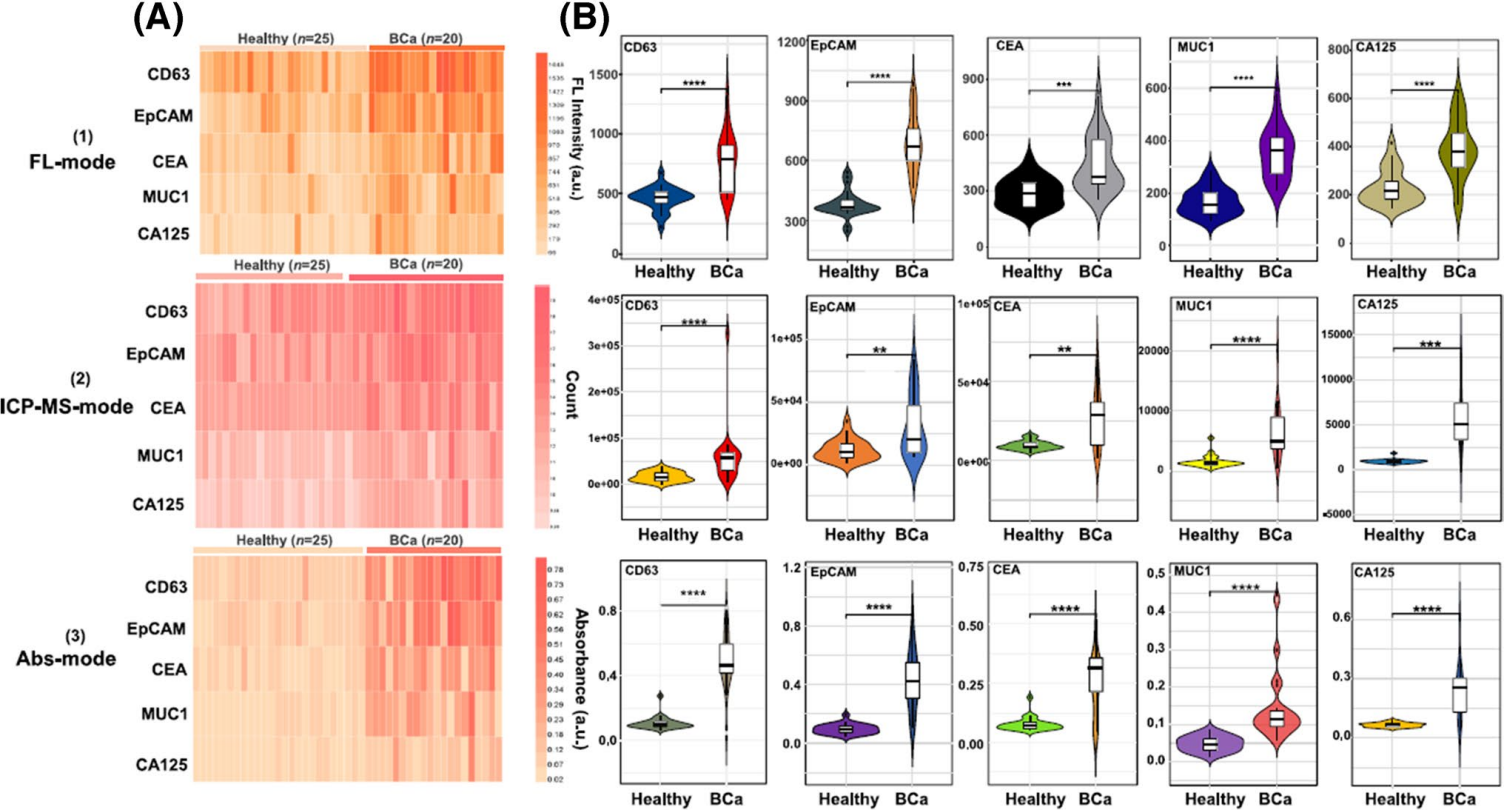
(**a**) Heatmaps of uEVs protein profiles from 25 healthy donors and 20 BCa patients in three types of detection modes. (**b**) Estimated average expression of CD63, EpCAM, CEA, MUC1, and CA125 in uEVs derived from healthy donors and BCa patients in three types of detection modes. Significant elevation of five protein expression levels on uEVs from BCa patients, as compared to healthy donors (Two-tailed two-sample *t* test was used to calculate the group differences. **p < 0.01, ***p < 0.001, ****p < 0.0001). Reproduced with permission from ref. [95]. Copyright (2024) American Chemical Society

Several multispectral 3D DNA machines (i.e. MB-based particles containing Ag nanoclusters, DNA aptamers and G-quadruplex/hemin) were applied for isolation of exosomes from urine and for subsequent analysis of presence of several receptors using three different spectral readings (Fig. 10) [95]. 3D DNA machine was constructed using one of the following aptamers: CD63 aptamer, EpCAM aptamer, MUC1 aptamer, CEA aptamer or CA125 aptamer for exosome isolation. Analysis of exosomes was performed by fluorescent, inductively coupled plasma mass spectroscopy and UV–Vis spectroscopy. Data were processed using machine learning algorithms. The approach was able to detect exosomes with LOD of 9.2 × 10^3^ exosomes/mL. Finally, the approach was applied for analysis of presence of several receptors on exosomal membranes (CD63, EpCAM, CEA, MUC1 and CA125) using all three detection methods. All three methods were evaluated to discriminate 20 bladder cancer patients from 25 HC with the following AUC values: 0.754–0.865 (fluorescent), 0.719–0.819 (MS), and 0.715–0.745 (colorimetric). When several biomarkers were combined for all three methods of detection the following discrimination power was obtained: 0.876 (colorimetric), 0.921 (MS) and 0.984 (fluorescent) (Fig. 11) [95].

**Fig. 12:**
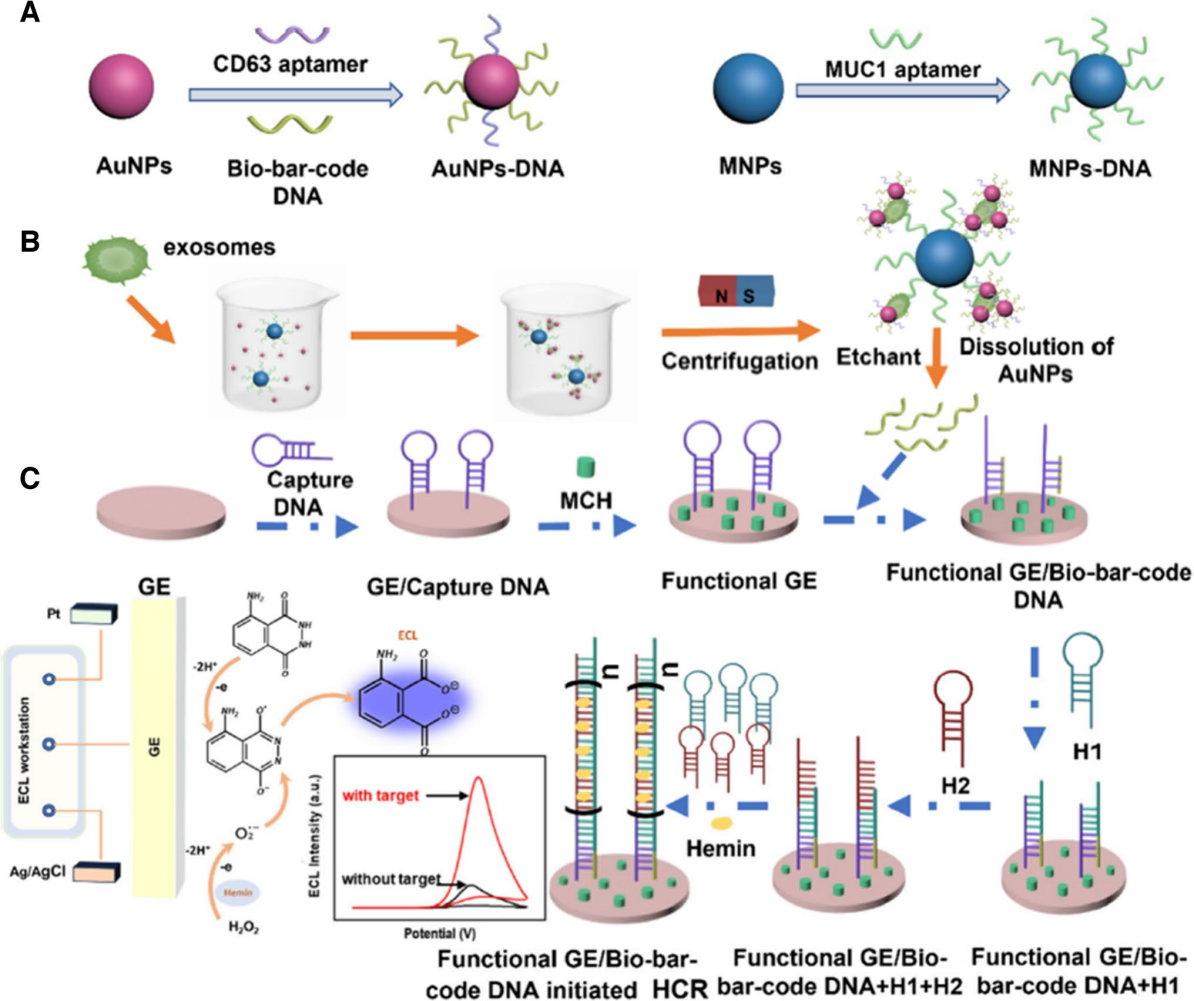
Construction of the ECL biosensor and its application in the sensitive detection of exosomes based on BCA and HCR-mediated dual signal amplification*a a* (**a**) Preparation procedure of AuNPs-DNA and MNPs-DNA nanoprobes. Ultrasensitive detection of exosomes through the DNA bio-bar-code (**b**) and HCR (**c**)-mediated dual signal amplification. Reproduced with permission from Ref. [96]. Copyright (2024) American Chemical Society

MBs modified by MUC1 aptamer were applied for isolation of exosomes and exosome detection was completed using a detection probe composed of AuNPs with CD63 aptamer and bio-bar-code DNA (Fig. 12) [96]. In the next step, AuNPs were dissolved and bi-bar-code released was detected with several DNA probes including hemin based on a hybridisation chain reaction (HCR)-mediated dual signal amplification protocol. Exosomes produced by MCF-7 cell line were detected with LOD of 5 × 10^3^ exosomes/mL using an ECL detection platform [96].

An ultra-sensitive and a simple electrochemical method for isolation and detection of exosomes was based on the combination of CRISPR/Cas12a, aptamer and MBs for the determination of BCa-derived exosomes [97]. MBs contained EpCAM aptamer to which a partially complementary DNA probe P1 was hybridised. Upon incubation with exosomes, P1 was released from MBs and combined with CRISPR RNA (crRNA), triggering the trans-cleavage activity of CRISPR/Cas12a, leading to removal a part of DNA probe P2 present on Au electrode containing methylene blue. In the absence of exosomes, this action of events did not occur with methylene blue still present on the Au electrode. Exosome produced by MCF-7 cell line were detected with LOD of 280 exosomes/mL using an electrochemical detection platform. When the approach was applied for detection of exosomes present in plasma samples from 10 HC and 10 BCa patients, it was possible to discriminate them with AUC = 1.000 [97].

Nanoboxes composed of CD63 aptamer were applied for isolation of exosomes and for their detection using three different modes, i.e. photothermal, colorimetric, and fluorescent [98]. Exosomes produced by human gastric cancer cells SGC-7901 were detected with LODs of 1.5 × 10^5^ exosomes/mL using a photothermal, 2.3 × 10^4^ exosomes/mL using a colorimetric and 5.8 × 10^3^ exosomes/mL using a fluorescent detection, respectively. When, the nanoboxes were applied for isolation of exosomes and further detection of exosomes as biomarkers, a significant discrimination between sera of HC (10) and gastric cancer patients (GC) (10) was observed with *p* < 0.001 [98].

An interesting approach based on implementation of lanthanide doped NPs exhibiting a weak magnetism was investigated recently [99]. MBs loaded with CD63 aptamer were used for selective capture of exosomes, to which lanthanide doped nanoparticles modified by CD63 aptamer were attached with aggregation of lanthanide doped nanoparticles in a confined space affecting their magnetic properties. A special device based on magnetic levitation sensing system called Maglev was developed to measure changes in the magnetic properties of aggregated particles. Exosomes produced by a HepG2 cell line were detected in the range from 7.5 × 10^4^ exosomes/mL to 1.5 × 10^7^ exosomes/mL. Blood samples from HC and BCa patients were discriminated with *p* ≤ 0.001 [99].

**Fig. 13:**
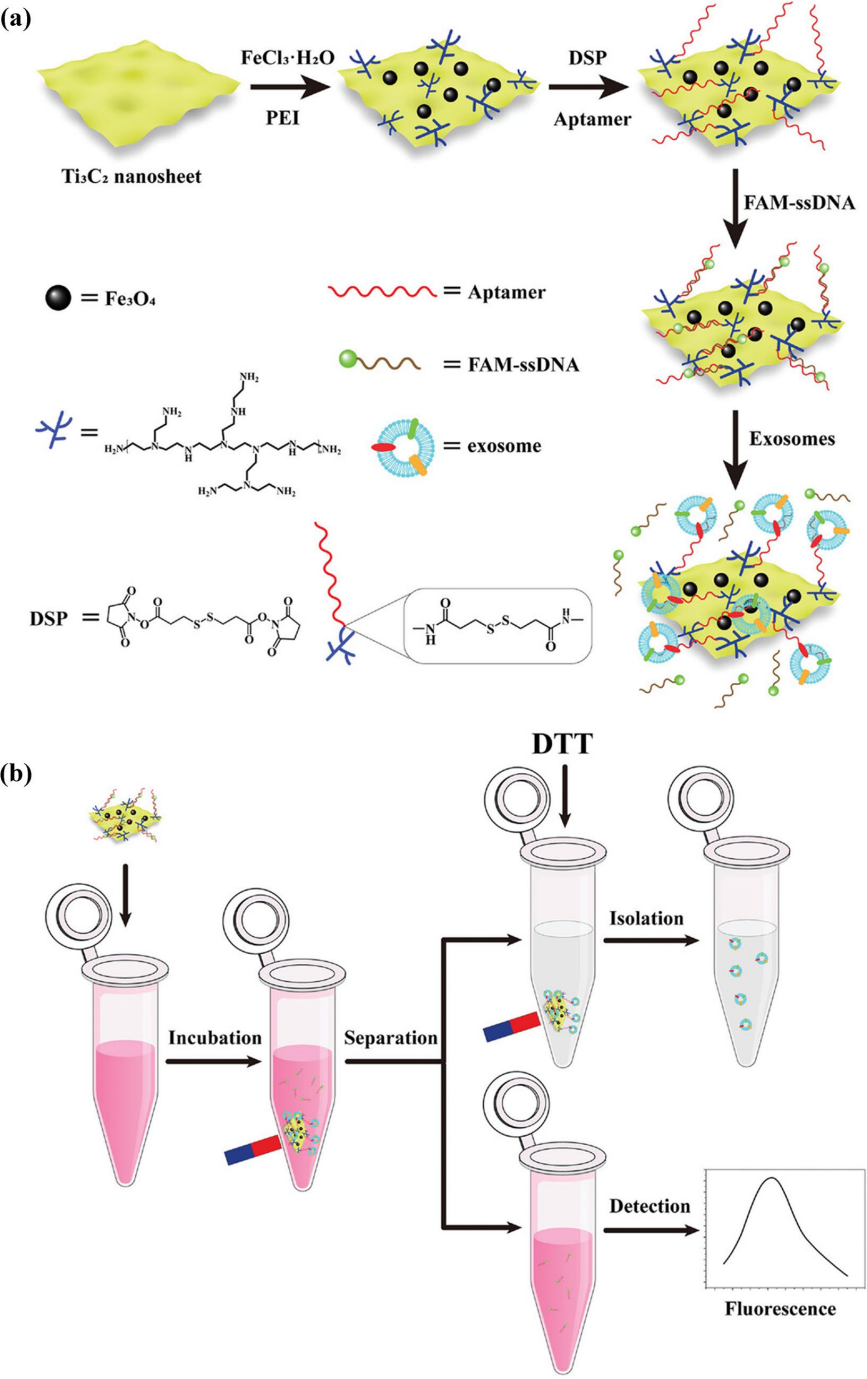
Schematic diagram of the preparation of Fe_3_O_4_@Ti_3_C_2_@PEI@DSP@aptamer@FAM-ssDNA, and the procedure for enrichment and detection of exosomes. Reproduced with permission from ref. [100]. Copyright (2024) John Wiley and Sons

A novel 2D nanomaterial MXene made of Ti_3_C_2_ nanosheets was used for accommodation of CD63 aptamer (conjugated to polyethyleneneimine and a disulphide linker) and MBs (Fig. 13) [100]. The aptamer was hybridised with a partly complementary ssDNA sequence having a fluorophore, which fluorescence was quenched by 2D nanomaterial. Upon incubation with exosomes, ssDNA labelled with a fluorophore was released and a fluorescent signal used for detection of exosomes was restored. A disulphide linker was reduced by DTT and exosomes were released from aptamer and could be used for further analysis. Exosomes produced by HepG2 cell line were detected with LOD of 4.2 × 10^4^ exosomes/mL [100].

MBs modified by CD63 aptamers were used for isolation of exosomes [101]. In the next step, MBs with exosomes attached were incubated with 2D nanomaterial MXene (2D nanomaterial) with immobilised EpCAM aptamer making a sandwich configuration with an enhanced electrochemically active area when deposited on the electrode. The electrochemical sensor was able to detect exosomes produced by various cell lines with LOD down to 4.3 × 10^4^ exosomes/mL [101].

**Fig. 14:**
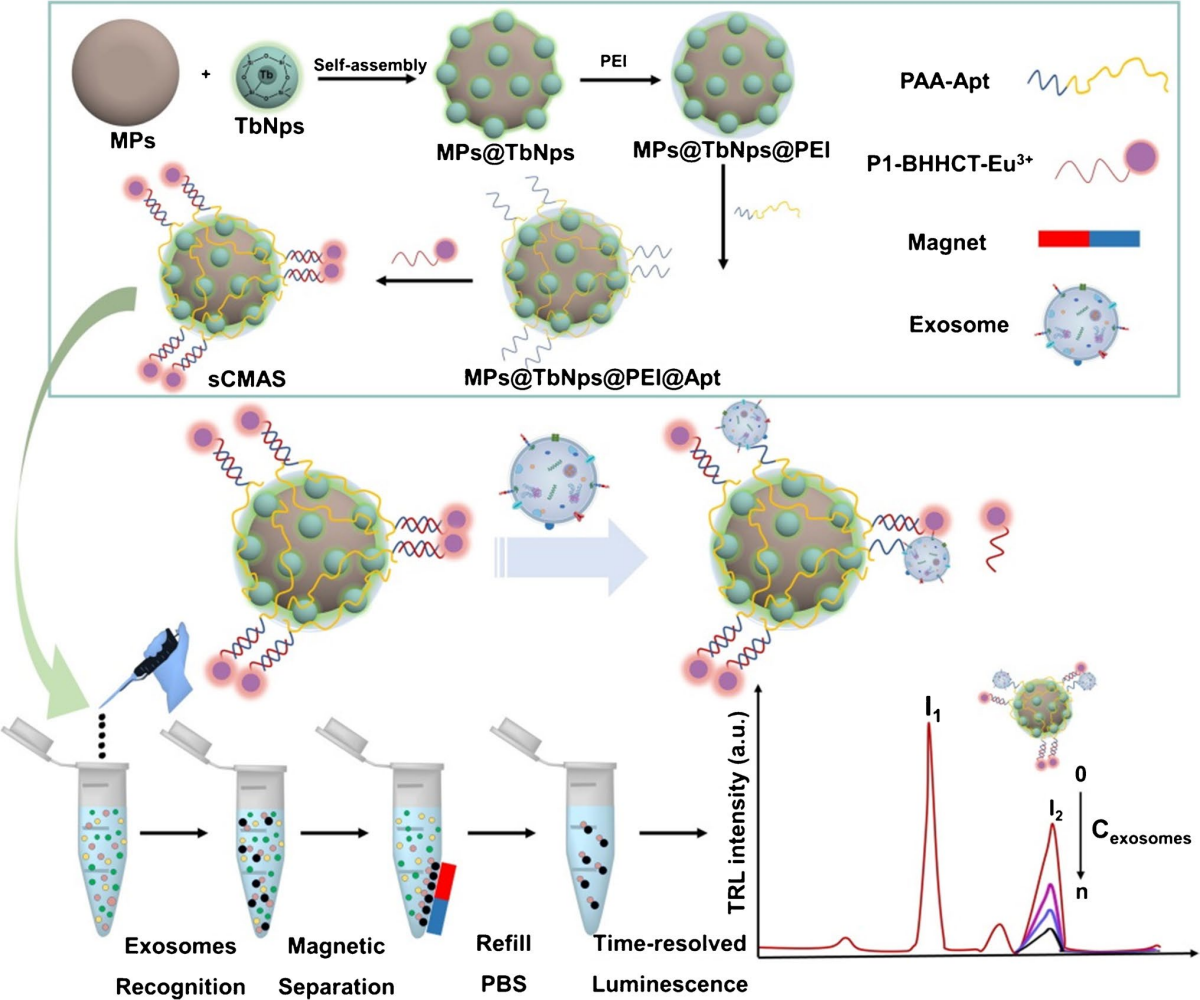
Illustration of the working principle for high-sensitive detection of melanoma exosomal PD-L1 based on sCMAS. Reproduced with permission from ref. [102]. Copyright (2023) Elsevier

A self-calibrating sensor was developed on MBs with two different fluorescent dyes present, i.e. Tb-based NPs attached to the surface of MBs, while Eu complex was attached to DNA sequence hybridised to PD-L1 aptamer (Fig. 14) [102]. Upon binding of exosomes, Eu complex was detached from the modified MB with a decrease in time-resolved luminescence. Such a decrease was relativized to the signal of Tb-based NPs. Exosomes produced by a mouse melanoma cell line (B16-F10) were detected with LOD of 190 exosomes/mL. Furthermore, the method was applied to discriminate HC from LCa patients with early metastasis (*p* < 0.0001), HC from LCa patients with late metastasis (*p* < 0.0001) and LCa patients with early metastasis from LCa patients with late metastasis (*p* = 0.0074) [102].

An interesting approach how to isolate and electrochemically detect exosomes was based on MBs modified by EpCAM aptamer and the electrode modified by a pH sensitive layer [103]. When EpCAM aptamer was bound to exosomes, additional form of DNA labelled with choline was added and only then biotinylated form of DNA could hybridise on choline-labelled DNA and DNA aptamer. In the next step, streptavidin-labelled glucose oxidase was added and able to produce gluconic acid and decreasing pH on the electrode. Decrease in pH triggered decomposition of a pH sensitive layer making the electrode surface more electrochemically active. Exosomes were isolated from two EpCAM-positive cell lines, i.e. SK-BR-3 and MCF-7 (BCa cell lines), and from two EpCAM-negative normal cell lines, i.e. MCF-10A and THP-1. Exosomes produced by SK-BR-3 cell line were detected with LOD of 2.2 × 10^4^ exosomes/mL. The approach was also applied for analysis of serum samples from HC (10) and BCa patients (10) before and after surgery with the signal obtained using serum samples from BCa patients before surgery higher that using serum samples from BCa patients after surgery [103]. Moreover, the signal from HC and BCa patients after surgery was quite similar, indicating that the approach could be used for monitoring of effectivity of surgery or for patient monitoring.

Detection of exosome was done by their isolation using MBs with immobilised CD63 aptamer followed by an incubation with a detection probe composed of a metal organic framework loaded with a hemin and PdNPs with peroxidase-like activity [104]. Exosomes produced by BCa cell line MCF-7 were detected with LOD of 8.6 × 10^4^ exosomes/mL [104].

A visual detection of exosomes was realised using MBs modified by CD63 aptamer for capture of exosomes with subsequent incubation with AuNPs modified by nucleolin aptamer [105]. The aptamer sequence was then extended by long polyT sequences to which AuNPs with attached polyA sequences were hybridised. An aggregation of both types of AuNPs was induced by an addition of NaCl with a signal generated detected by colorimetry, but also by a naked eye. Exosomes produced by a human leukaemia cell line HL-60 were detected with LOD of 4.5 × 10^4^ exosomes/mL [105].

**Fig. 15:**
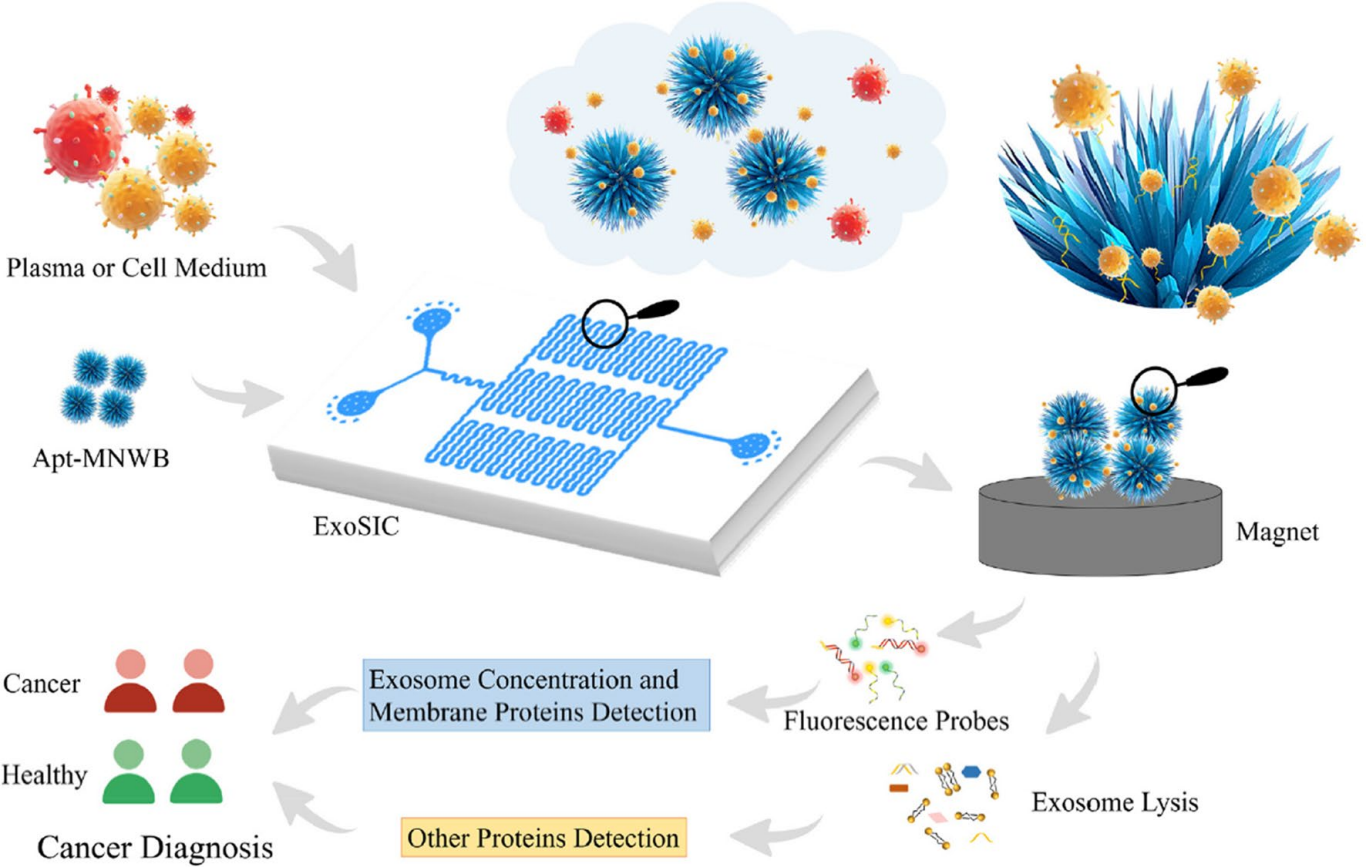
Workflow of the ExoSIC chip for the continuous isolation of circulating exosomes and cancer diagnosis. Reprinted with permission from ref. [106]. Copyright (2023) Royal Society for Chemistry

Continuous exosome isolation in the microfluidic format was possible using magnetic nanowaxberry modified by CD63 aptamer (Fig. 15) [106]. The device was able to isolate exosomes with 24-fold higher yield compared to DUC with a purity higher compared to a precipitation method and a high specificity. Finally, the approach was used for isolation of exosomes from plasma samples of HC (25) and LCa patients (23) with ability to discriminate them with AUC = 0.725, when exosome concentration together with presence of exosomal EGFR and EpCAM receptors was considered [106].

**Fig. 16:**
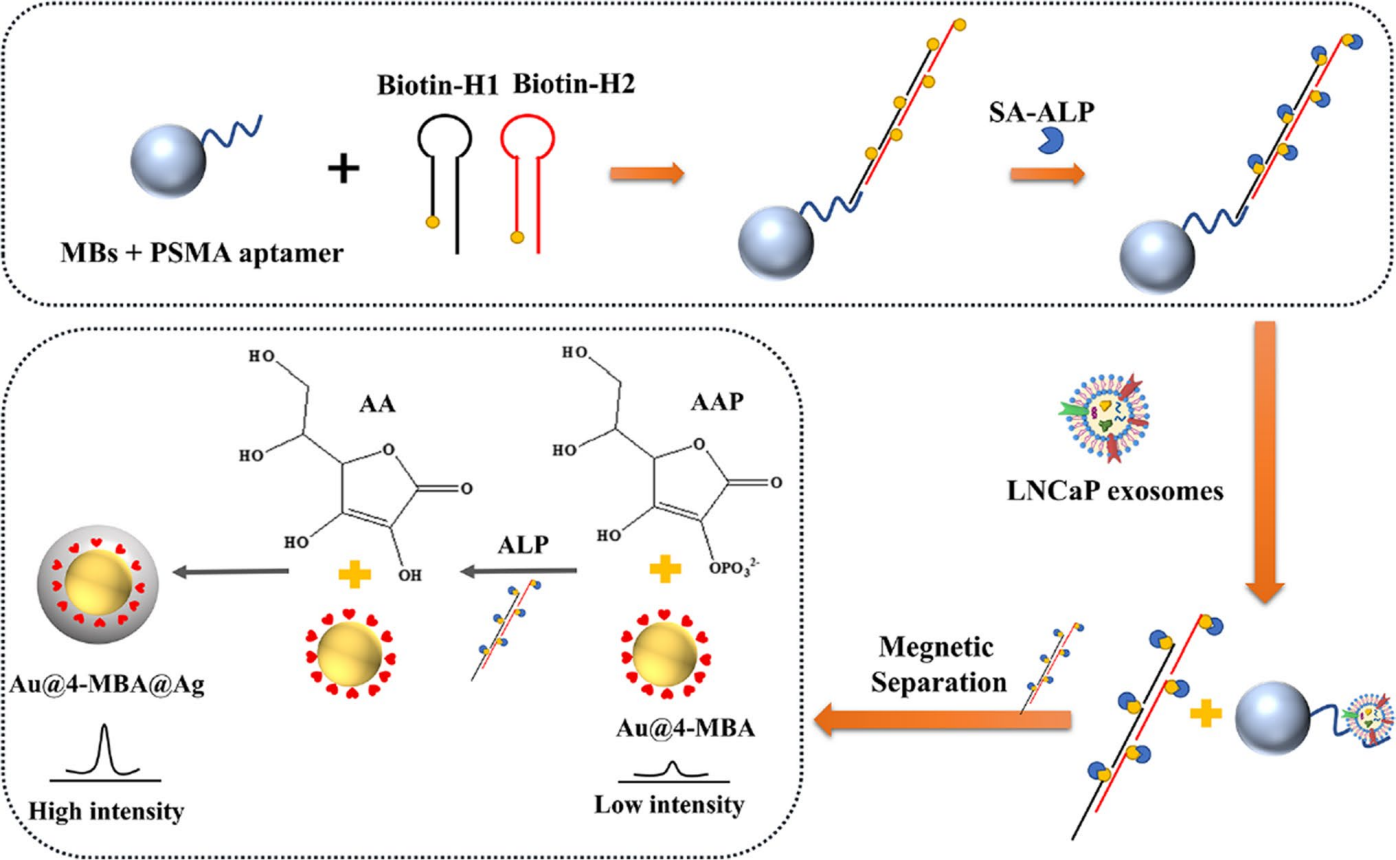
Schematic of the aptamer-induced HCR for the rapid SERS immunoassay of exosomes. Reproduced with permission from ref. [107]. Copyright (2023) American Chemical Society

An interesting approach using a hybridised chain reaction-amplified chain integrating alkaline phosphatase (ALP)-induced formation of Ag-shell nanostructure on the modified MB was applied for detection of exosomes (Fig. 16) [107]. MB modified with PMSA (prostate specific membrane antigen) aptamer was hybridised with a biotinylated probe H1 and H2. Upon incubation with a streptavidin–alkaline phosphatase conjugate, numerous enzyme molecules were delivered along dsDNA chain containing also aptamer sequence. After capture of exosome, a long dsDNA sequence with multiple enzymes was detached from MBs and released into the solution. Such an enzymatic conjugate was then reacting with modified AuNPs resulting in deposition of an Ag shell on the AuNPs with a subsequent generation of a SERS signal. Exosomes produced by a prostate cancer cell line LNCaP were detected with LOD of 1.9 × 10^4^ exosomes/mL. The sensor was also applied for analysis of serum samples from HC (9) and PCa patients (10) with a significant discrimination power represented by *p* = 0.0041 [107].

### Lessons learned from aptamer-based AC of exosomes

The simplest strategy for determination of exosome concentration is to use 1st aptamer attached to MBs with the 2nd aptamer attached to some tag to generate the signal (i.e. a SERS [42], a colorimetric [105] or a magnetic signal [99]).

Aptamers used for AC of exosomes allow several strategies for generation of signal, what is not possible using antibody-based AC of exosomes. Rolling circle amplification (RCA) is one of such strategies, when using this approach it is possible to insert various tags in multiple copies along DNA chains, i.e. fluorescent dyes [86], enzymes (peroxidase [87] or alkaline phosphatase [91]), or several AuNPs-based probes [88]. Hybridisation chain reaction (HCR) can be also applied for amplification of the signal, i.e. by for insertion of multiple hemins into the DNA sequences (Fig. 12) [96] or by insertion of multiple enzyme molecules of alkaline phosphatase (Fig. 16) [107]. Another strategy behind a signal amplification using aptamers include use of a DNA walker [41] or 3D DNA machine [95].

Similarly to approaches described for Ab-based AC of exosome isolation, also for aptamer-based AC of exosomes two subpopulations of exosomes could be detected using three different aptamers (1st one for exosome isolation and 2nd and 3rd for visualisation of exosome sub-populations) [86]. A similar approach was used for determination of 5 subpopulation of exosomes [95] and in one paper the authors described even use of seven different aptamers attached to MBs, which could be used to determine concentration of 7 different sub-populations of exosomes within the same sample [93].

MBs [41] or a hybrid material composed of MBs and 2D nanomaterial [100] could be also applied as quenchers of fluorescence with fluorescence restored after release of a fluorescent dye from the surface of MBs or hybrid MB-particles.

Specificity of exosome analysis could be achieved by combination of two different affinity probes (i.e. probe 1 containing aptamer 2 and probe 2 aptamer 3) for specific detection of only exosomes expressing both receptors [88].

While for Ab-based AC of exosomes quite strong agents need to be used to release exosomes from MBs modified by Abs, in case of aptamer-based AC of exosomes, exosomes could be released using near infrared irradiation changing the tertiary structure of an aptamer [85] or using complementary DNA strands having strong affinity towards aptamers [89]. Such released exosomes can be then used for analysis of exosomal cargo for example by a MS analysis [89].

Although in principle determination of exosome concentration is possible using a ratiometric approach for Ab-based AC of exosomes, it was so far described only for aptamer-based AC of exosomes. The method is based on the use of MBs modified by one type of a tag (i.e. a fluorescent tag 1) and upon incubation with exosomes another tag is introduced onto MBs (i.e. fluorescent tag 2) and the signal from fluorescent tag 2 is relativized to fluorescent tag 1 providing ratiometric signal improving assay performance [90]. Similar approaches were used for generation of a ratiometric luminescent signal in two studies [92, 102].

Exosomes still attached to aptamer modified MBs were directly deposited on target plate for Laser Desorption Ionisation MS analysis of exosomes, what significantly simplified MS analysis of exosomes [94].

There is only one example when aptamers and antibodies were combine for determination of exosomes using MBs modified by aptamers with a signal tag delivered to exosomes by antibody interaction with exosomal receptors [93].

It seems that aptamer-based AC of exosomes is really a flexible format for determination of exosomes since there are two studies showing that the same assay format allows three different types of a signal reading [95, 98].

A novel technology based CRISPR/Cas-12a system was also applied for determination of concentration of exosomes [97].

Finally, aptamer-based AC of exosomes can be made more specific by a combined interaction of probes with exosomes either using cholesterol modified DNA [103] or using Zr-metal–organic framework using Zr(IV) to interact with phosphate groups of phospholipids of exosomes [104].

### Other biorecognition elements for AC of exosomes

**Fig. 17:**
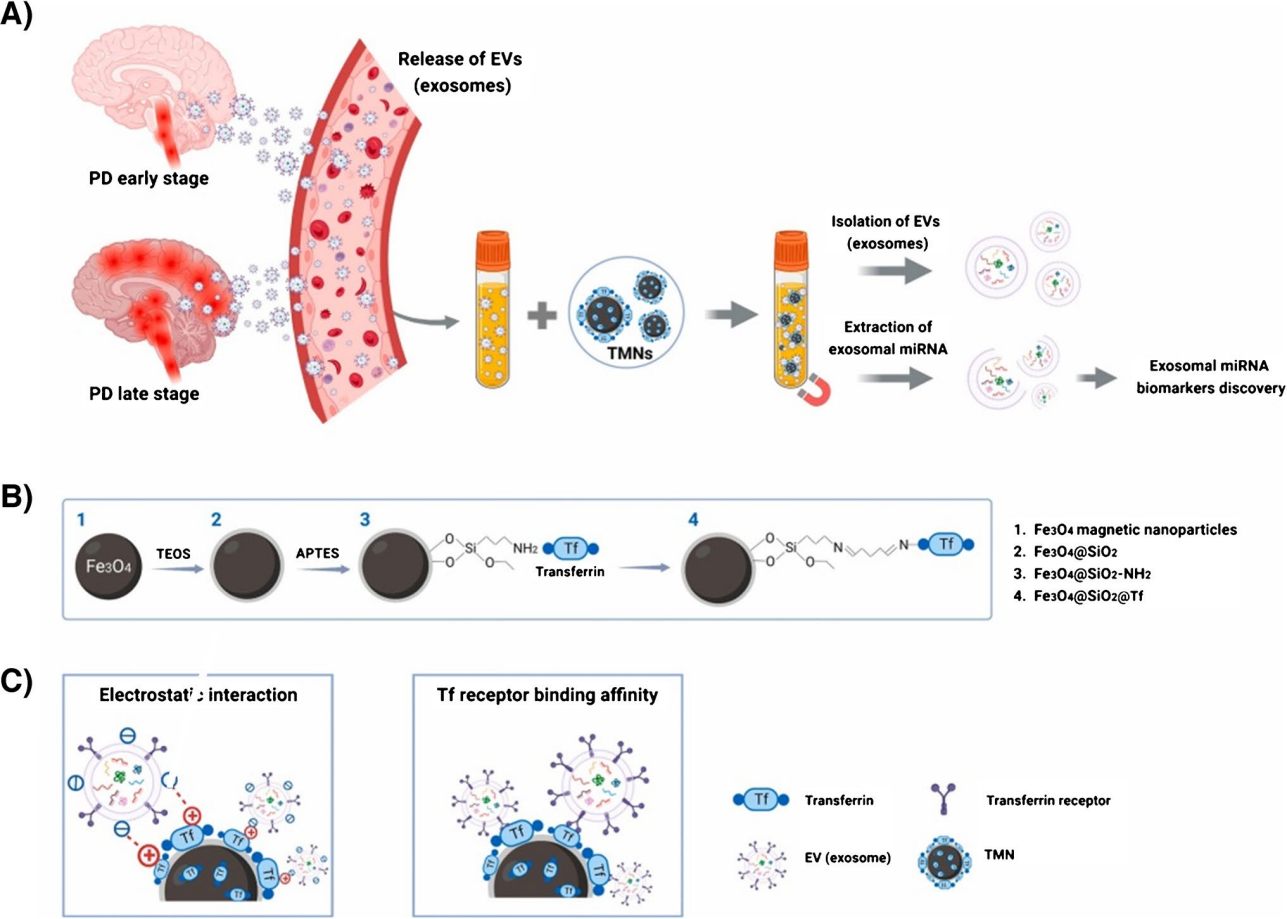
Schematic representation and application of the TMNs assay for EV isolation. (**a**) Schematic depiction of the EV isolation process from human plasma specimens utilizing the TMNs assay. (**b**) Schematic illustration of the TMNs synthesis. (**c**) Mechanisms of electrostatic interaction (left) and ligand-receptor interaction (right) between TMNs and EVs. This image was created using BioRender (https://biorender.com). TMNs: transferrin magnetic nanoparticles; EV: extracellular vesicles; Tf: transferrin. Reproduced with permission from Ref. [49]. Copyright (2024) Elsevier

Transferrin modified MBs were applied for isolation of brain-derived exosomes with the dual-mode interaction based on electrostatic attraction between negatively charged exosomes and positively charged transferrin-modified MBs or using presence of transferrin recognised receptors on exosomal surface (Fig. 17) [49]. In the next step, miRNA was analysed in isolated exosomes, which was applied for a biomarker discovery. Analysis was performed using 5 plasma samples from HC; 25 plasma samples from humans (12 Parkinson disease (PD) patients at an early stage and 8 Parkinson patients at a late stage), 4 patients with dementia and 4 patients with multiple sclerosis . In such samples 8 different exosomal miRNAs were measured with six of them (miR-195-5p, miR- 495-3p, miR-23b-3P, miR-30c-2-3p, miR-323a-3p, and miR-27a-3p) markedly upregulated in PD patient plasma, when comparing to HC [49]. A similar approach for brain-derived exosome isolation based on transferrin-modified MBs was used for detection of peptide fingerprinting as biomarkers using LC–MS [50]. The study identified 21 protein differently expressed in exosomes present in serum samples of patients having three different neurological diseases (PD, dementia and multiple sclerosis) compared to HC [50], which could be used for diagnostic purposes [50].

### Peptide-based AC of exosomes

Phage display technology was applied for identification of peptides binding to exosomes [46]. The best identified peptide C3 immobilised on MBs was then applied for exosome isolation from several BCa cell lines (MCF-7, MDA-MB231 and SKBr3) and a control cell line (MRC-5). Magnetic colorimetric ELISA using biotinylated peptide C3 (combined with streptavidin-polyHRP) was then used for exosome detection with LOD in the range 3.3–4.7 × 10^5^ exosomes/mL depending on a cell line. An electrochemical signal readout offered LOD in the range 1.7–2.6 × 10^5^ exosomes/mL depending on cell line [46].

### (Phospho)lipid-based AC of exosomes

MBs coated with a tetrahedral lipid probe were used for enrichment of exosomes [108]. In the next step PD-L1 aptamer was attached to the surface of exosomes and initiated a catalytic hairpin assembly with a subsequent formation of H1/H2 duplexes. Such duplexes activated CRISPR/Cas12a, which cleaved a DNA probe containing a fluorescent dye and a quencher and a fluorescent signal was restored. The sensor could detect exosomes produced by non-small cell lung cancer cell line NCI-H1975 in an ultrasensitive way with LOD down to 1.7 × 10^6^ exosomes/mL. Plasma samples from 10 HC and 20 non-small cell lung cancer patients were analysed with this approach with a discrimination power represented by AUC of 0.890 compared to ELISA offering AUC of 0.760 [108]. A similar approach based on activation of CRISPR/Cas12a and release of a fluorescent dye from DNA conjugated with a fluorescent dye was used for detection of exosomal miRNA-21, which was able to discriminate blood samples from HC and LCa patients with AUC of 1.000 [109].

MBs modified with imprinting layer were used for affinity interaction with phospholipids of exosomes [43]. A hybrid AuNPs modified by anti-CD9 antibodies were then applied for interaction with CD9 receptors present on exosomes. Exosomes from urine of healthy individual were detected using SERS with LOD of 5.8 × 10^7^ exosomes/mL [43].

Selection of the rare earth elements strongly interacting with phosphate groups revealed europium as the best one [110]. Core–shell MBs were then prepared using Eu_2_O_3_ present in the outer layer for specific interaction with phosphate groups for isolation of exosomes. The method was 1.5-fold more effective in isolating exosomes compared to UC and was applied for isolation of exosomes from plasma samples of patients with hepatocellular carcinoma (HCC). Finally, analysis of metabolites in exosomes was performed with identification of 70 differently expressed metabolites when comparing HCC with control cohorts with chenodeoxyglycocholate, hexylresorcinol, capsiamide were the most up-regulated metabolites [110].

**Fig. 18:**
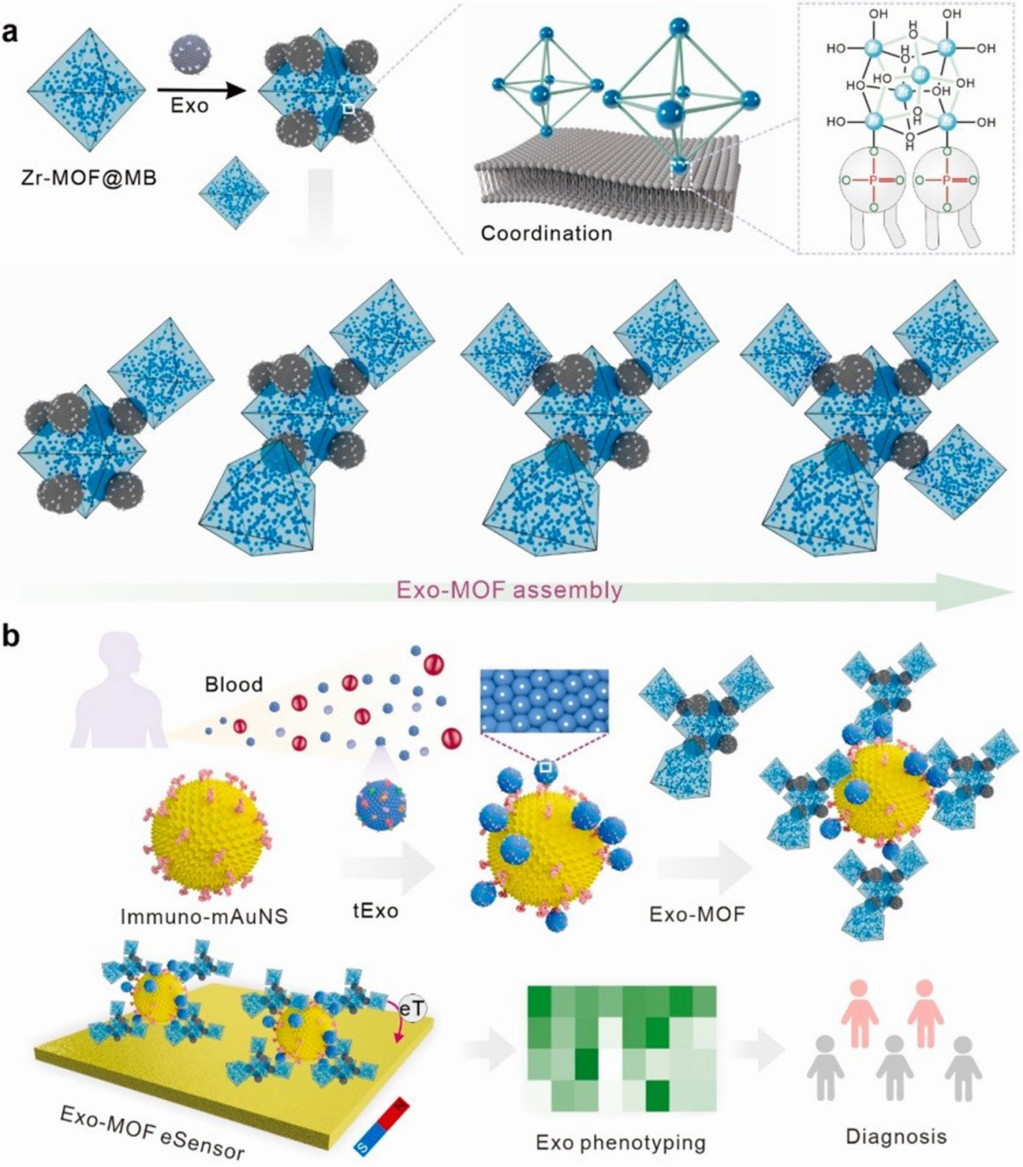
Overview of Exo-MOF eSensor for Exo phenotyping and cancer diagnosis. (**a**) Schematic illustration of tunable assembly of Exo-MOF signal amplifier via Zr-phosphate coordination. (**b**) Schematic of engineering Exo-MOF eSensor for Exo phenotyping and cancer diagnosis from clinical blood samples by coupling immuno-magnetic Au nanostars (immuno-mAuNSs) with an Exo-MOF signal amplifier. Reproduced with permission from ref. [111]. Copyright (2024) Elsevier

**Fig. 19:**
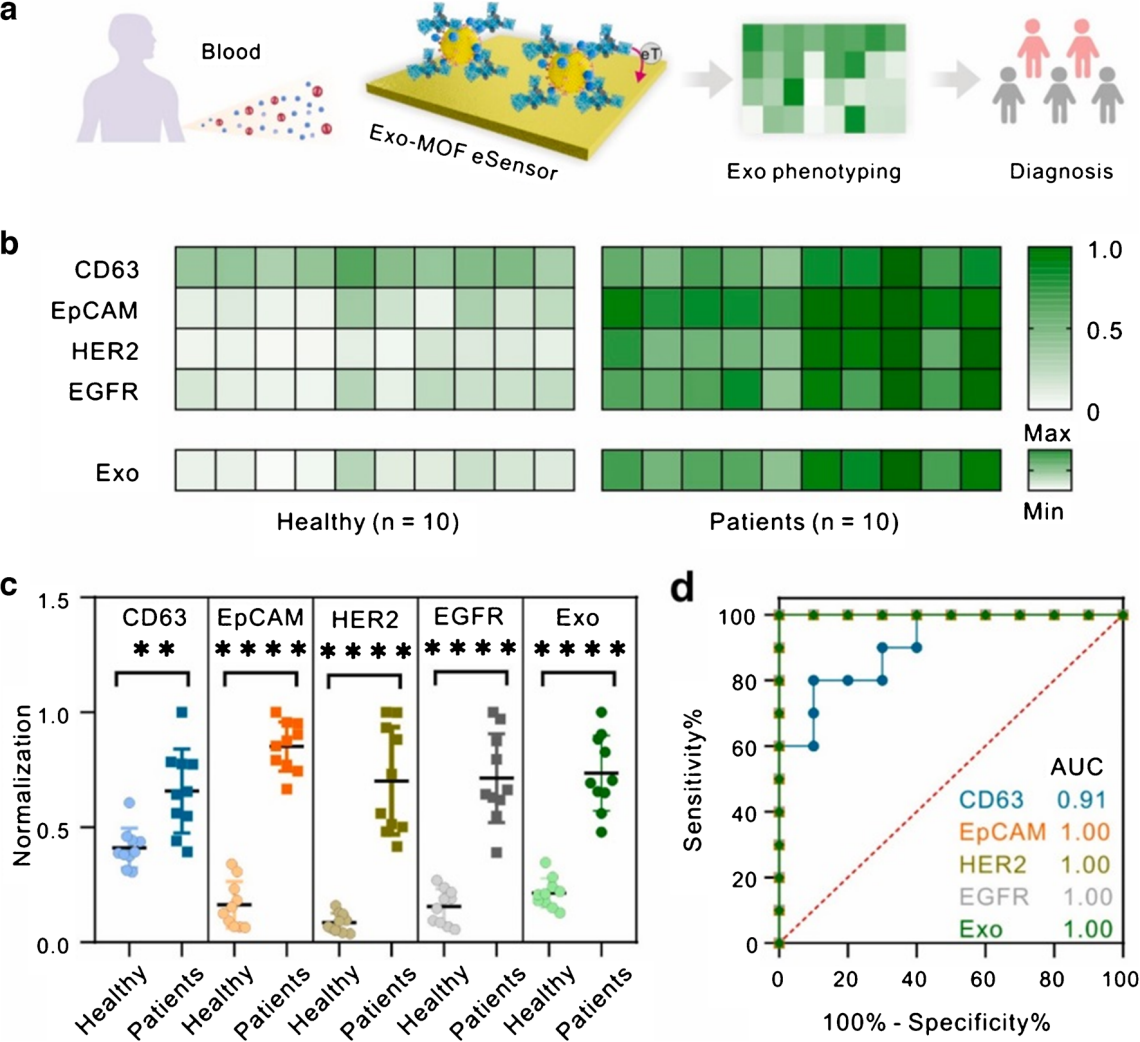
Exo-MOF eSensor for clinical diagnostics application. (**a**) Schematic of phenotypic detection of target Exo derived from human plasma for clinical diagnosis. (**b**) Heatmap of the relative expression level of four exosomal protein markers (CD63, EpCAM, HER2, and EGFR) on plasma Exo estimated by normalized signal change and their weighted sum (S_Exo_). (**c**) Scattering plots of the normalized levels of four exosomal protein markers and their weighted sum (S_Exo_) in comparison between the healthy donors and breast cancer patients. (d) ROC curve analysis evaluating the diagnostic accuracy of four individual exosomal protein markers and the S_Exo_ to discriminate patients from the healthy controls. **p < 0.01, ****p < 0.0001 (Student’s *t*-test). Error bar means ± sd (*n* = 3). Reproduced with permission from ref. [111]. Copyright (2024) Elsevier

Zr-modified metal organic framework (MOF) was applied for interaction of exosomes via phospholipids (Fig. 18) [111]. MBs covered with AuNPs in a shape of nanostars were used for immobilisation of antibodies against exosomal receptors, i.e. CD63, EpCAM, HER2, and EGFR and then incubated with MOF-exosome conjugates to probe presence of specific receptors on the surface of exosomes. Since MOF caged redox active molecules, when the whole complex was delivered to the electrode surface, an electrochemical signal reading was possible. The sensor was used for analysis of exosomes produced by several cell lines such as MCF-7, MDA-MB-231, HepG2, A-431, HeLa, A549, RAW264.7, SK-BR-3, and MCF-10A. When using hybrid MBs modified by Anti-EpCAM antibody for detection of exosomes produced by MCF-7 cells, the sensor could detect exosomes down to LOD of 10 exosomes/mL. Finally, the human blood plasma samples of HC (10) and BCa patients (10) were analysed with the sensor with extremely high discrimination power with AUC in the range from 0.91 to 1.00 (Fig. 19) [111].

**Fig. 20:**
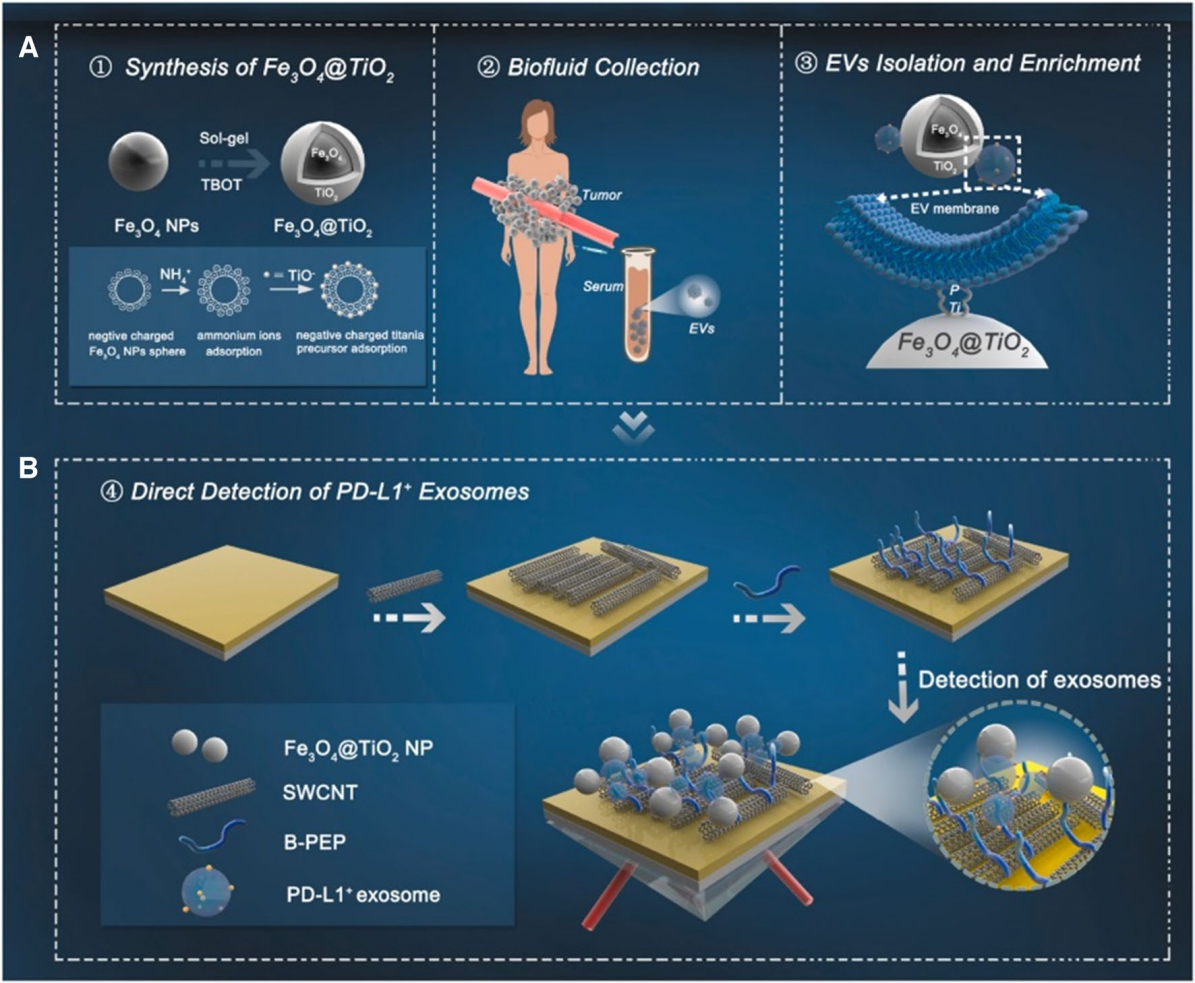
Schematic diagram of the synthesis of Fe_3_O_4_@TiO_2_ and its coordinate-based selective enrichment with exosomes (**a**), and the SWCNT-enhanced SPR sensor with Fe_3_O_4_@TiO_2_ signal amplification for detection of PD-L1^+^ exosomes (**b**). Reproduced with permission from ref. [44]. Copyright (2024) Elsevier

MBs modified by a TiO_2_ outer layer were used for exosome isolation via an interaction with phospholipids (Fig. 20) [44]. Exosomes captured on modified MBs were then injected over the SPR surface modified by single walled carbon nanotubes and a peptide sequence recognising PD-L1 receptor present on the exosomal surface. The device offered LOD of 32 exosomes/mL produced by the cell line MDA-MB-231. The SPR sensor was also used for analysis of human serum samples from BCa patients and HC with excellent discrimination power represented by AUC = 0.984 [44].

MBs functionalised using Ti-metal framework were integrated for isolation of exosomes via an interaction with phospholipids [45]. Then, isolated exosomes attached to modified MBs were transferred to MALDI-TOF plate for analysis of exosomal metabolites, when Ti-metal framework worked also as an ionisation matrix. The protocol was then tested to identify disease biomarkers (metabolites with M_w_ in the range from 100 Da to 1,000 Da) by analysis of 238 serum samples (119 from HC, 110 from Alzheimer disease (AD) patients and 9 from PD patients). Eight different algorithms were then used for data analysis with subsequent identification of a presence of 377 unique metabolites in such samples. Using all different algorithms taken into account, 377 metabolites with a discrimination power to distinguish AD from HC and PD patients with the best AUC = 1.0. Finally, three metabolites, including allantoic acid, L-tyrosine and butyrylcarnitine were identified as AD-specific biomarkers showing AUC between 0.980 and 1.000 [45].

Exosomes were isolated from complex samples using SEC and in the next step MBs modified by a TiO_2_ layer were used for attachment of purified of exosomes produced by a pancreatic cell line PANC-1 via a phospholipid affinity interaction on modified MBs [112]. A specific targeting of glypican-1 present on exosomes was done using AuNPs modified with anti-glypican-1 antibody with presence of mass tag (m/z = 695 Da). In order to make MALDI-TOF MS analysis quantitative, AuNPs modified with internal standard (m/z = 783 Da) were also analysed. Exosomes could be analysed in the range from 7.1 × 10^4^ to 7.1 × 10^9^ exosomes/mL. Furthermore, the assay was also applied for analysis of serum samples of HC (11) and PaCa patients (34) with p < 0.0001. When PaCa patients were divided into three categories depending on the size of tumour from small (PaCa1), medium (PaCa2) to large (PaCa3), there were differences according to the *t* test result between HC and PaCa1; HC and PaCa2, HC and PaCa3, together with PaCa2 and PaCa3 were significant, corresponding to *p* < 0.001, *p* < 0.0001, *p* < 0.0001, and *p* < 0.01, indicating that exosomal glypican-1 expression can be used for monitoring of PaCa progression and/or treatment prognosis. The authors also calculated AUC values for discrimination PaCa *vs.* HC (0.957) and PaCa1 *vs.* PaCa2 (0.895) [112].

### Glycan-based AC of exosomes

Concanavalin A (lectin, i.e. a glycan recognising protein) modified MBs were applied for isolation of exosomes from urine using lectin-glycan interaction [48]. While UC was able to isolate (5.7 ± 0.6) × 10^10^ exosomes from 50 mL of urine, MB-based method was able to isolate (1.2 ± 0.1) × 10^10^ exosomes from 50 mL of urine. The method was finally used for analysis of miRNA-21 in urine sample from one BCa patient and one healthy donor with a minor difference between these two samples [48].

Modified MBs by a layer of hydrophilic polymers wasere applied for isolation of exosomes through glycoproteins present on their surface from urine samples [113].

Here it is worth mentioning that some approaches for exosome isolation claimed by affinity towards phospholipids of exosomes (as specified in the previous section) might be also via interaction of modified MBs with glycans present on exosomes. A good example is a TiO_2_ layer which offers strong interaction with glycans.

### Other methods of AC for exosomes

An electrostatic method for isolation of exosomes was based on application of MBs entrapped in the positively charged self-assembled nanoclusters interacting with negatively charged exosomes [114]. Exosomes can be eluted from MBs using 1 M NaCl solution to screen the charges on exosomes and nanoclusters. Exosomes isolation efficiency of the approach was much better compared to the commercially available exosome purification kit ExoQuick and PEG-based exosome isolation and exosome isolation procedure can be repeated using the same nanoclusters [114].

Magnetic 3D ordered macroporous zeolitic imidazolate framework-8 was synthesized and applied for robust isolation of exosomes from urine [115]. Exosomes were attached to the composite (actually resembling imprinted polymers) using size exclusion, phospholipid affinity and electrostatic interactions. Analysis required 50 mL of urine and isolation of 42 urine samples was completed in 2 h. Machine learning algorithms were applied for data treatment of data obtained by analysis of exosomal metabolites using LC–MS/MS reveal identification of 797 metabolites. The approach was able to discriminate early-stage bladder cancer (BC) patients (12) from HC (16) with AUC in the range 0.844–0.997 with retinol esters as the best biomarkers [115].

**Fig. 21:**
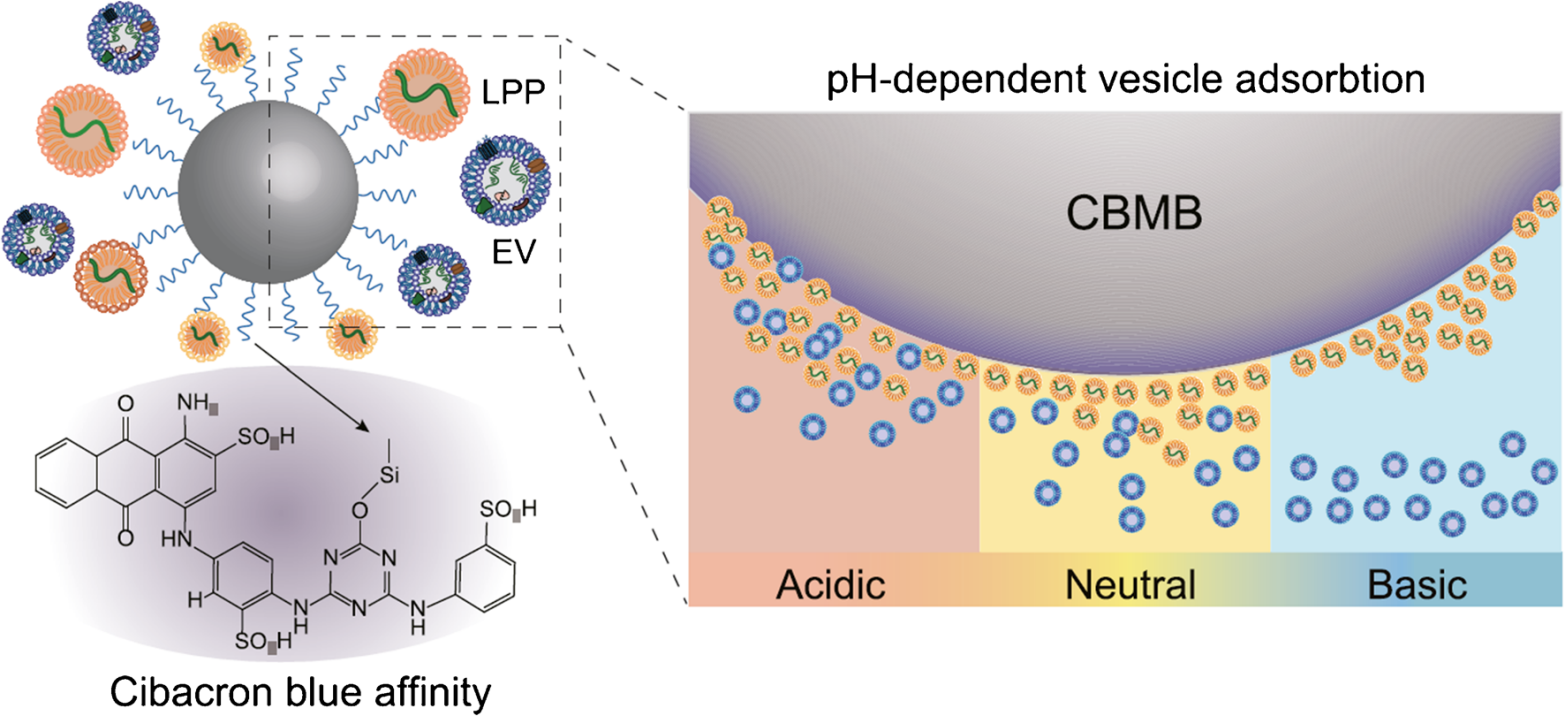
The pH-mediated separation of extracellular vesicles (EVs) and lipoproteins (LPPs). Reproduced with permission from ref. [116]. Copyright (2023) Elsevier

A simple way for separation of exosomes from lipoproteins was based on modification of MBs with cibacron blue (Fig. 21) [116]. While lipoproteins are attached to MBs with cibacron blue at acidic, neutral and basic conditions, exosomes are bound to such modified MBs only at acidic and neutral pH. Thus, basic conditions can be used for affinity capture of lipoproteins on MBs modified with cibacron blue, while exosomes stay in the solution and could be separated in other way [116].

## Conclusions

The main drawback of AC-based methods for exosome isolation, i.e. high cost caused by a high cost of antibodies applied was addressed in many papers and from the overview presented in this review paper. It is now clear that there is an increasing number of studies replacing antibodies by other (bio)affinity ligands especially by aptamers with several advantages compared to antibodies. DNA/RNA aptamers offer high affinity towards their analytes with K_D_ down to pM level and at the same time offering high selectivity [118]. Additionally, aptamers are able to discriminate between small structural differences in the structure of analyte such as enantiomeric configuration [118]. Defined chemical structure, size, and synthetic production of aptamers overcome significant disadvantages of antibodies as biorecognition molecules. Furthermore, a limited “richness” of functional groups available using standard nucleotides can be enhanced using nucleotide derivatives or derivatization of prepared aptamers [119]. Finally, aptamers offer another interesting feature, i.e. allow a signal amplification using various strategies such as rolling circle amplification, hybridization chain reaction, strand-displacement reaction, etc. [120]. Such a feature is essential for isolation and analysis of subpopulations of cell-type specific exosomes circulating in blood at low concentrations. A detailed comparison of advantages and disadvantages for using antibodies and DNA/RNA aptamers is provided in a recent review paper [121].

There are other affinity-based ligands, which can be applied for isolation of exosomes such as phospholipid affinity-based protocols, transferrin-based (for isolation of brain-derived exosomes), peptides, lectins (or other glycan-recognition approaches) and other approaches based on imprinted polymer or electrostatic interactions. The vast majority of AC-based methods of isolation of exosomes are based on integration of antibodies or aptamers or in few cases their combination (see Sects. 4.1 and 4.3). There are only limited number of studies describing use of other alternative (bio)affinity ligands for AC-based isolation of exosomes either integrating peptides, imprinted polymers, determination of glycans, using other affinity ligands like phopsholipids or relying on other affinity interactions, which are rather non-specific like electrostatic ones. The main reason behind that fact might be that such approaches are not that specific and/or with high affinity constants like for antibody and aptamer based approaches. This is why it is good to combine other alternative (bio)affinity ligands with highly specific ligands including antibodies and/or aptamers to really achieve high specificity of isolation of exosomes.

It is advised that AC-based exosome isolation methods are combined with other isolation techniques such as SEC, what can result in more robust exosome isolation protocols.

Table 5 shows that there are already available methods able to detect really low concentration of exosomes down to tens of exosomes/mL and that all methods described in Table 5 are sensitive enough with LOD well within or below the concentration of exosomes present in serum/plasma samples. What is even more encouraging in the fact that most of the AC-based methods of exosome isolation offered LOD well below estimated concentration of exosomes expected to be released into blood stream from tissues/organs (~ 10^7^ exosomes*/*mL). Thus, isolation of tissue/organ specific exosomes is really feasible, what was documented in many cases by analysis of biofluids (saliva, urine and blood serum/plasma). Furthermore, in many cases a significant discrimination power of exosomes (expressing specific receptors) was declared either using *p*-test analysis (*p* < 0.0001) or using ROC analysis with AUC values within range from 0.900 to 1.000. There are however only 2 studies using more than 100 samples used in the clinical validation study. Thus, there is very important to confirm very high discrimination power of several preliminary approaches as listed in Table 5 by clinical validation studies using hundreds of clinical samples. Such large validation studies could be performed only when the analytical methods used for exosome detection and analysis of exosomal cargo are well characterised using analytical validation studies. The focus in such analytical validation studies should be on analytical precision, accuracy, reproducibility of the assays; inter-day and intra-day assay variability, variability caused by different operators in the same laboratory and variability of the assays in different laboratories.

SERS was quite frequently used for detection of exosomes (Table 5), but there is also a new emerging technique applicable for analysis of exosomes, i.e. Surface-Enhanced Infrared Absorption (SEIRA) spectroscopy based on utilisation of plasmonic metasurface for immobilisation of (bio)affinity ligands [122]. The method allows reading mid-infrared signatures with enhanced sensitivity compared to a conventional infrared spectroscopy [123], what is a key attribute for achieving high sensitive as specific exosome-based diagnostics.

An interesting conclusion can be drawn from two studies using several detection platforms while using the same isolation protocol. There seems to be a correlation between high analytical sensitivity of the method (represented by low LOD) and a discrimination power of the approach (represented by *p*-value or AUC value). This preliminary finding needs to be proved in other studies, but if this assumption will be confirmed it will mean that it is very important in further developments to focus on isolation/detection methods using exosomes or exosomal content with high analytical sensitivity (low LOD values).

There is one study showing that a simple method based on MB-modified by cibacron blue could be used to supress isolation of lipoprotein particles together with exosomes [116], especially based on affinity capture of phospholipids since concentration of phospholipids in blood is few orders of magnitude higher compared to exosomes.

Most of the studies presented in Table 5 were focused on analysis of the concentration of exosomes used as disease biomarkers and there are only a few studies focused on analysis of exosomal content, i.e. exosomal metabolites and proteins as disease biomarkers. For the latter case it would be however very important to develop highly sensitive methods of analysis to quantify a subtle amount of biomolecules present in exosomes, as was recently suggested using optimised enzymatic protein digestion and chromatography gradient length [124].

We expect that the number of studies integrating magnetic AC-based isolation and subsequent analysis of exosomes and exosomal content will exponentially increase with identification of really robust disease biomarkers clinically verified in clinical studies using hundreds of samples for the future benefit of patients.

## Data Availability

No datasets were generated or analysed during the current study.
